# Fiber-rich diet with brown rice improves endothelial function in type 2 diabetes mellitus: A randomized controlled trial

**DOI:** 10.1371/journal.pone.0179869

**Published:** 2017-06-29

**Authors:** Keiko Kondo, Katsutaro Morino, Yoshihiko Nishio, Atsushi Ishikado, Hisatomi Arima, Keiko Nakao, Fumiyuki Nakagawa, Fumio Nikami, Osamu Sekine, Ken-ichi Nemoto, Makoto Suwa, Motonobu Matsumoto, Katsuyuki Miura, Taketoshi Makino, Satoshi Ugi, Hiroshi Maegawa

**Affiliations:** 1 Department of Medicine, Shiga University of Medical Science, Otsu, Shiga, Japan; 2 Department of Public Health, Shiga University of Medical Science, Otsu, Shiga, Japan; 3 Department of Diabetes and Endocrine Medicine, Kagoshima University Graduate School of Medical and Dental Sciences, Kagoshima, Japan; 4 R&D Department, Sunstar Inc., Takatsuki, Osaka, Japan; 5 Department of Preventive Medicine and Public Health, Faculty of Medicine, Fukuoka University, Fukuoka, Japan; 6 Osaka Laboratory, CMIC Pharma Science Co., Osaka, Japan; Universita degli Studi di Milano, ITALY

## Abstract

**Background & Aims:**

A fiber-rich diet has a cardioprotective effect, but the mechanism for this remains unclear. We hypothesized that a fiber-rich diet with brown rice improves endothelial function in patients with type 2 diabetes mellitus.

**Methods:**

Twenty-eight patients with type 2 diabetes mellitus at a single general hospital in Japan were randomly assigned to a brown rice (*n* = 14) or white rice (*n* = 14) diet and were followed for 8 weeks. The primary outcome was changes in endothelial function determined from flow debt repayment by reactive hyperemia using strain-gauge plethysmography in the fasting state. Secondary outcomes were changes in HbA_1c_, postprandial glucose excursions, and markers of oxidative stress and inflammation. The area under the curve for glucose after ingesting 250 kcal of assigned rice was compared between baseline (T0) and at the end of the intervention (T1) to estimate glucose excursions in each group.

**Results:**

Improvement in endothelial function, assessed by fasting flow debt repayment (20.4% *vs*. −5.8%, p = 0.004), was significantly greater in the brown rice diet group than the white rice diet group, although the between-group difference in change of fiber intake was small (5.6 g/day *vs*. −1.2 g/day, p<0.0001). Changes in total, HDL-, and LDL-cholesterol, and urine 8-isoprostane levels did not differ between the two groups. The high-sensitivity C-reactive protein level tended to improve in the brown rice diet group compared with the white rice diet group (0.01 μg/L *vs*. −0.04 μg/L, p = 0.063). The area under the curve for glucose was subtly but consistently lower in the brown rice diet group (T0: 21.4 mmol/L*h *vs*. 24.0 mmol/L*h, p = 0.043, T1: 20.4 mmol/L*h *vs*. 23.3 mmol/L*h, p = 0.046) without changes in HbA_1c_.

**Conclusions:**

Intervention with a fiber-rich diet with brown rice effectively improved endothelial function, without changes in HbA1c levels, possibly through reducing glucose excursions.

## Introduction

Previous studies have shown that intake of dietary fiber decreases the risk for cardiovascular events [1, 2]; however, the molecular mechanisms underlying the cardioprotective effects of dietary fiber remain unknown. Difficulties in proving the cardioprotective effects of fiber are related to the long duration between exposure and the cardiovascular event as well as multiple confounders. Few dietary intervention studies have assessed the causality of this phenomenon [3].

Endothelial dysfunction is a potent predictor of future cardiac events in patients with hypertension and in older adults [4, 5]. Improved endothelial function associated with medications such as statins may play a part in the cardioprotective mechanism of these medications [6]. Dietary intervention with a Mediterranean diet improves endothelial function [7]. The Mediterranean diet comprises fruit, vegetables, fish, and whole grains, with limited unhealthy fats, and is known to reduce cardiovascular events [3]. Recently, we reported that a fish-based dietary intervention improved endothelial function in patients with type 2 diabetes mellitus (T2DM) [8], a disease that is a known risk factor for endothelial dysfunction. We hypothesized that a fiber-rich dietary intervention may also improve endothelial function, and considered that improvement of endothelial function is a good surrogate marker of further cardiovascular events [9].

Fiber consumption varies by individual, but, in general, is not sufficient in the U.S. and Japan [10, 11]. The American Diabetes Association recommends an intake of 14 g of fiber per 1000 kcal for individuals with T2DM [12]. A high-fiber diet has been reported to improve postprandial glucose excursions [13], systemic inflammation [14], and dysregulation of adipocytokines [15]. Each of these factors may attenuate endothelial dysfunction in patients with T2DM.

Rice is a staple food of more than half of the world’s population. Through refining processes, the outer bran and germ portions of intact rice grains (i.e., brown rice) are removed to produce white rice, which primarily comprises starchy endosperm. As a result, white rice contains about five times less fiber than brown rice [16]. Therefore, a diet can be fiber enriched by consuming brown rice instead of white rice. Here, we hypothesized that a fiber-rich diet with brown rice improves endothelial function in patients with T2DM through reduction of postprandial glucose, oxidative stress, and/or systemic inflammation. To this end, we examined the effects of a fiber-rich diet with brown rice compared with consumption of a white rice diet on endothelial function in patients with T2DM.

## Materials and methods

The full protocol for this trial and supporting CONSORT checklist are available as supporting information (see S1–S5 Files). After the original protocol had been approved, additional analyses including asymmetric dimethylarginine (ADMA) were added as secondary outcomes for further mechanistic discussion. A change was also made to the rice tolerance test, in which participants were asked to eat 250 kcal instead of 150 g of the assigned rice for the diet-load. These modifications were applied before participants were randomized.

### Study design

**Fig 1:**
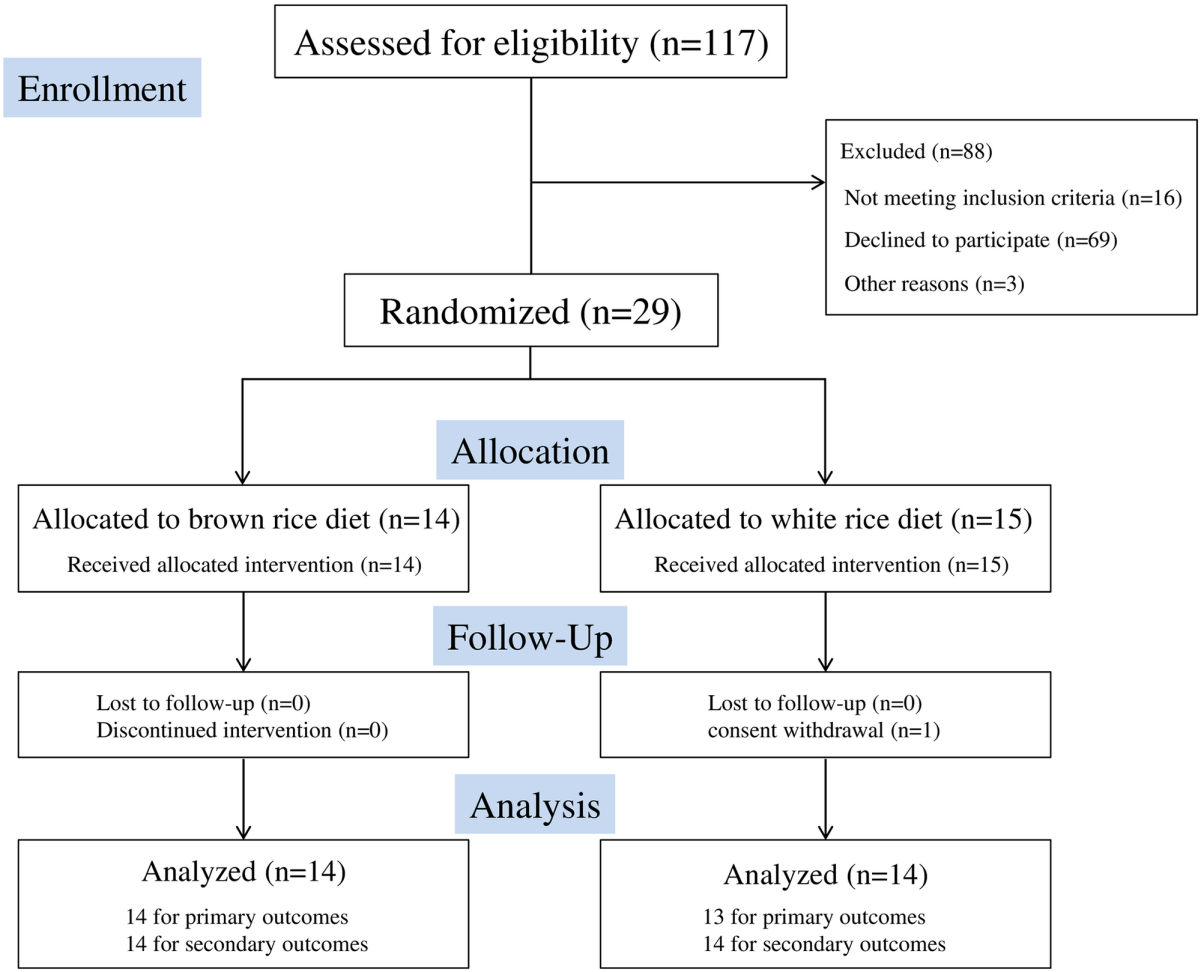
Flow chart showing the recruitment of participants.

**Fig 2:**
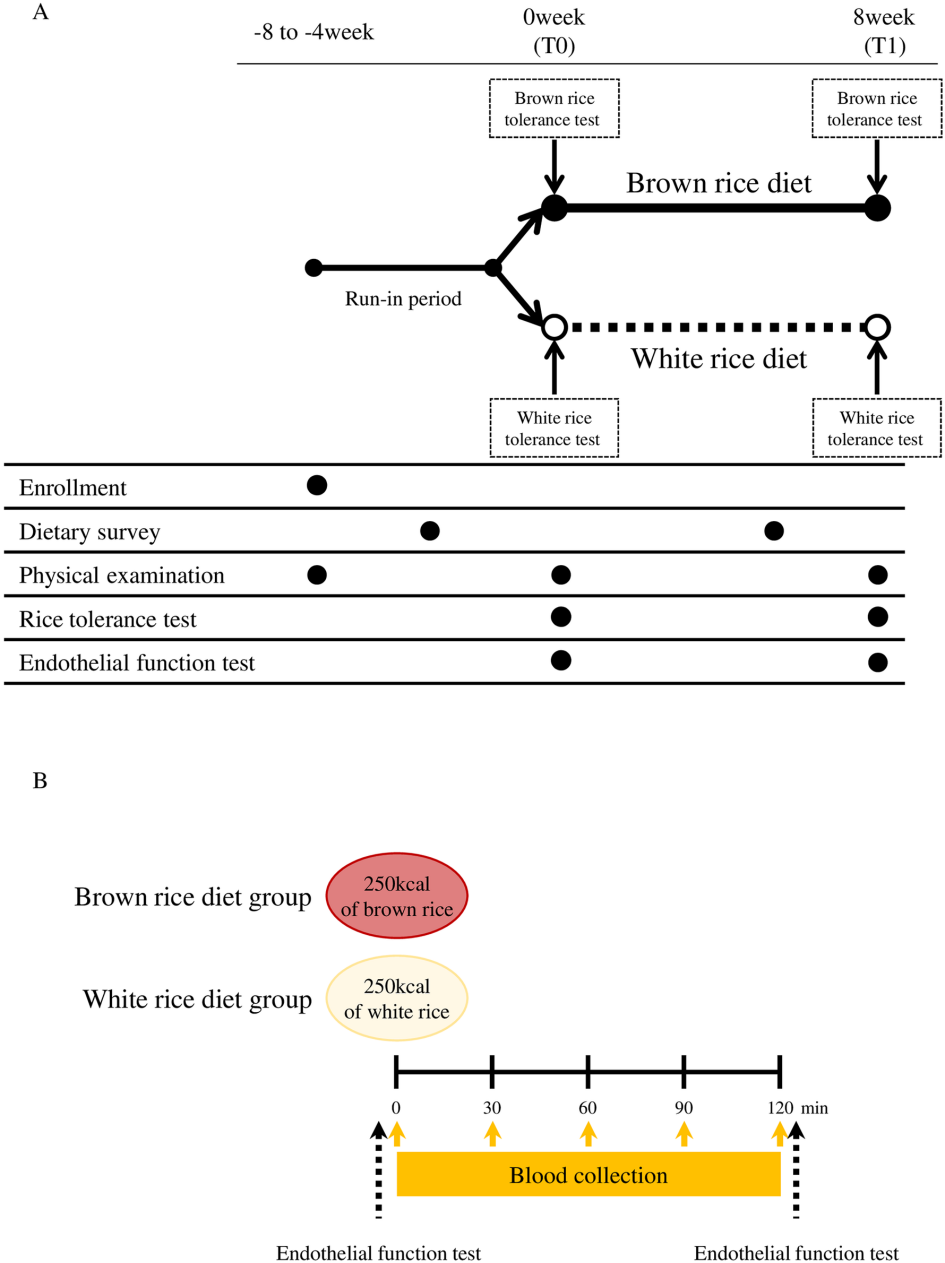
**(A) Study design**. An overview of the study design. **(B) Rice tolerance test**. A rice tolerance test was performed at baseline (T0) and at the end of intervention (T1). The participants in each group ingested an isocaloric (250 kcal) amount of assigned rice. Blood collections were performed at fasting, 30, 60, 90, and 120 min after assigned rice ingestion. Endothelial function tests were performed at fasting and 120 min after assigned rice ingestion.

The study was a randomized, open-labeled, parallel-controlled trial to investigate the effects of a fiber-rich diet with brown rice on endothelial function in patients with T2DM (Figs 1 and 2A).

### Study population

Patients with T2DM were invited to participate at Shiga University of Medical Science Hospital, Shiga, Japan from April 3, 2012 to April 1, 2014. The final date of follow up was December 9, 2014. Inclusion criteria were patients with T2DM aged between 40 and 80 years and with HbA_1c_ levels of less than 8.4%. Individuals taking an α-glucosidase inhibitor, consuming brown rice or dietary fiber supplements, undergoing insulin treatment, who had changed medications for T2DM within the previous 2 months, who were current smokers, who were pregnant or breastfeeding, or who had severe vascular, hepatic, renal, or infectious diseases or cancer were excluded. The nature and potential risks of the study were explained to all participants, and written informed consent was obtained. The study was performed in accordance with the principles contained within the Declaration of Helsinki. The protocol was approved by the Ethics Committee of Shiga University of Medical Science originally on September 27, 2011. The first modifications were approved on February 21, 2012 and the second modifications were approved on April 24, 2012. The study is registered at UMIN Clinical Trials Registry (http://www.umin.ac.jp/ctr/index.htm) with the Identification No. UMIN000008370. The authors confirm that all ongoing and related trials for this intervention are registered. Registration to UMIN occurred on July 6, 2012 because preparation for UMIN was delayed until receiving official documentation from the Institutional Review Board.

### Run-in period

Eligible patients underwent a 4- to 8-week run-in period to maintain a stable diet and assess tolerability. Participants were advised by a dietician to have a standard diet for diabetes, comprising 28–30 kcal/kg of ideal bodyweight (protein 15%, fat 25%, carbohydrate 60%) [17] at the beginning of the run-in period.

### Randomization

Randomization was stratified by age, sex, and body mass index (BMI). We used the minimization method for randomization. The number of strata for each factor was two. The cut off values for stratification factors were an age of 66 years and a BMI of 24. After the run-in period, participants were randomly assigned to a brown rice or white rice diet. Investigators were provided with a random allocation sequence made by a research assistant, who was independent of the investigators, using computer-generated random digits.

### Intervention

A feasible intervention protocol for a clinical setting was chosen for both groups. Both brown rice (for the fiber-rich diet) and white rice were provided in small single-serve pouches for the 8-week study to limit energy intake (provided by Sunstar Inc., Osaka, Japan). Barley and amaranth were blended with brown rice by the company to adjust the viscosity and flavor. In the brown rice diet group, participants were instructed to consume brown rice as a staple food for 10 out of 21 meals per week and to maintain their energy intake at 28–30 kcal/kg for 8 weeks. Similarly, in the white rice diet group, participants were instructed to consume white rice as a staple food for 10 out of 21 meals per week and to maintain their energy intake at 28–30 kcal/kg for 8 weeks. The nutritional components of the rice in the pouches are shown in S1 Table. Participants were advised to maintain their usual lifestyle habits, such as physical activity, throughout the study.

### Outcomes

The primary outcome was change in endothelial function assessed with fasting flow debt repayment (FDR). Changes in other endothelial functions (peak forearm blood flow (FBF) and duration of reactive hyperemia (RH)) in the fasting state were key secondary outcomes. Other secondary outcomes were changes in postprandial endothelial function (FDR, peak FBF, and duration of RH), nutritional intake, bodyweight, body fat, plasma levels of glucose and insulin in the fasting and postprandial states, HbA_1c_, glycoalbumin, triglyceride, total, high-density lipoprotein (HDL)- and low-density lipoprotein (LDL)-cholesterol, adiponectin, ADMA, tissue plasminogen activator inhibitor-1 (tPAI-1), high-sensitivity C-reactive protein (hs-CRP), and urine levels of 8-isoprostane.

### Procedures

#### Weight and body composition

The bodyweight of each participant, without shoes and with light clothing weighing a maximum of 0.1 kg, was recorded using an electronic scale. The percentage of body fat was determined by bioelectrical impedance analysis (Bio Electrical Impedance Analyzer System; Tanita, Tokyo, Japan).

#### Dietary assessment

During the run-in and intervention periods, a dietician provided participants with written and verbal instructions regarding the completion of a 3-day dietary record with a digital photograph of each meal. Nutritional intake was calculated using food composition tables specific for Japanese people (Eiyoukun, ver. 6.0; Kenpakusya, Tokyo, Japan) [16]. For processed foods, the nutritional status was obtained from the websites of food manufacturers, and available nutritional information was used for calculations.

#### Laboratory analyses

Blood samples were taken after an overnight fast except for meal tolerance tests, at both baseline (T0) and the end of intervention (T1). Plasma glucose and serum insulin levels were measured using the hexokinase glucose 6-phosphate dehydrogenase ultraviolet method and a chemiluminescent enzyme immunoassay, respectively. Serum triglyceride and total cholesterol levels were determined enzymatically and by the cholesterol dehydrogenase ultraviolet method, respectively. Serum HDL-cholesterol levels were determined by a direct method. LDL-cholesterol levels were calculated using the Friedewald equation (total cholesterol − [HDL-cholesterol + triglyceride/5]). Insulin resistance was evaluated using the homeostasis model assessment and calculated as the product of fasting glucose and insulin levels. HbA_1c_ levels were measured by the latex agglutination method. Glycoalbumin levels were determined enzymatically. Serum total adiponectin (Otsuka Pharmaceutical, Tokyo, Japan) and ADMA (Immundiagnostik AG, Bensheim, Germany) levels were measured by ELISA. Serum tPAI-1 levels were measured by latex photometric immunoassay. Serum hs-CRP levels were measured by nephelometry. Urine 8-isoprostane levels were measured by enzyme immunoassay.

#### Rice tolerance test

A rice tolerance test was performed to evaluate plasma levels of glucose and insulin at T0 and T1 after an overnight fast (Fig 2A). Because the aim of the rice tolerance test was the estimation of the postprandial state of each group during the intervention, participants in each group ingested an isocaloric (250 kcal) amount of brown or white rice. Blood samples were collected before and at 30, 60, 90, and 120 min after assigned rice ingestion. After fasting and 120 min, endothelial function was determined by RH using strain-gauge plethysmography (Fig 2B). The nutritional composition of both brown rice and white rice is shown in S1 Table.

#### Endothelial function

The endothelial function of each participant was examined after an overnight fast and at 2 h postprandial state after ingestion of their assigned rice. Each participant was kept in the supine position during this measurement. After 15 min in the supine position, basal FBF was measured using a mercury-filled Silastic (Dow Corning, Midland, MI, USA) strain-gauge plethysmograph (EC-6; D.E. Hokanson, Issaquah, WA, USA), as described by Linder *et al*. [18]. The effect of RH on FBF was measured as described in other studies, with minor modifications [8, 19]. To induce RH, FBF was occluded by inflating the cuff on the right upper arm to a pressure of 190 mmHg when the systolic blood pressure (SBP) was ≤140 mmHg, or 50 mmHg plus SBP when the SBP was >140 mmHg, for 5 min. FBF was measured after release of the cuff until it returned to the basal level. The peak FBF response [18], duration of RH, and total reactive hyperemic flow (FDR) were used to assess the resistance of vessel endothelial function. The percent peak FBF, an index of the peak FBF response, was obtained by calculating the increment of the peak FBF divided by the mean basal FBF. FDR was obtained by calculating the area under the curve (AUC) of the increment of FBF response after RH was divided by the AUC of the basal FBF (S1 Fig) [20]. The reproducibility of FDR measurements in our laboratory and inter-day coefficients of variation was 6.1%.

### Statistical analysis

We calculated our sample size using results from a preliminary study, in which intervention with a brown rice diet increased FDR by 83.1 ± 60.2% and a white rice diet increased FDR by 19.5 ± 40.9% (unpublished data). We estimated that, with a sample size of 30, the study would have 80% power to detect differences in the outcome between randomized groups, assuming an overall lost-to-follow-up rate of 14% with a type 1 error of 5% (two-sided).

Statistical analyses were performed according to the principle of intention-to-treat using SAS (ver. 9.4; SAS Institute Inc., Cary, NC, USA). Data are expressed as mean ± SD for continuous variables and *n* (%) for categorical variables. The primary outcome of change in fasting FDR was compared using an unpaired *t*-test between the brown rice and white rice diet groups. Similarly, changes in secondary outcomes were also compared using unpaired *t*-tests between randomized groups. In addition, we also analyzed the primary outcome using the Wilcoxon test. The levels of glucose and insulin during the rice tolerance test were compared using mixed model analysis. For sensitivity purposes, the primary outcome was analyzed after adjustment for baseline variables (age, BMI, SBP, fasting glucose, fasting insulin, total cholesterol, triglyceride, hs-CRP, tPAI-1, and adiponectin). A p-value <0.05 was considered statistically significant. No adjustment for multiplicity was planned because a single primary outcome was investigated.

## Results

### Participants and diet diaries

In total, 117 patients with T2DM were recruited at Shiga University of Medical Science Hospital between April 2012 and April 2014. Following a detailed explanation of the study, 29 patients were enrolled (Fig 1). After exclusion of one patient who withdrew her consent, 28 patients were included in the analysis. Because of the difficulty in measuring endothelial function in one patient who had a resting tremor, 27 patients were included in the analysis of the primary outcome, while 28 patients were included in the analysis of the secondary outcomes (Fig 1). The baseline characteristics were balanced between the two groups (Table 1). No participant changed their medications during the study and no adverse events were observed throughout the study period.

**Table 1:** Characteristics of participants at baseline.

	Brown rice group (n = 14)	White rice group (n = 14)
M/F	9/5	9/5
Age (years)	65.2±8.7	68.1±6.8
Medication		
Glucose-lowering agents, n (%)	11 (78.6)	14 (100)
Lipid-lowering agents, n (%)	5 (35.7)	8 (57.1)
Anti-hypertensive agents, n (%)	3 (21.4)	4 (28.6)
Duration of diabetes (years)	16.3±10.1	14.2±7.3

Values are mean ± SD for continuous variables. Mean (±SD) of eGFR at enrollment was 76.6 ± 17.2 mL/min/1.73 m^2^. M, male; F, female.

### Nutritional intake at baseline and during the intervention

Nutritional intake was analyzed at baseline and during the intervention period (Table 2). The intake of total energy and macronutrients was unchanged throughout the study period in both groups. The intake of micronutrients such as vitamin B_1_ and magnesium was also unchanged in both groups. Compared with the white rice diet group, intake of total dietary fiber significantly increased during the intervention period in the brown rice diet group (−1.2 g/day *vs*. 5.6 g/day; a difference of 6.8 g/day [95% CI for the difference, 4.2–9.3 g/day]).

**Table 2:** Nutritional intake at baseline and during the dietary intervention.

	Brown rice group			White rice group			P value
	Baseline	During the intervention	Change	Baseline	During the intervention	Change	
Energy (kJ/day)	7611±1731	7828±1633	216 (−369 to 829)	7437±1154	7552±966	116 (−407 to 639)	0.790
(kJ/kg)	123±34	127±27	4 (−6 to 13)	115±18	117±19	3 (−5 to 10)	0.836
Protein (%kcal)	16.3±1.6	15.8±2.1	−0.5 (−1.9 to 0.8)	16.8±1.5	16.1±1.8	−0.7 (−1.8 to 0.4)	0.884
Fat (%kcal)	25.5±4.4	27.2±5.7	1.8 (−1.6 to 5.1)	24.6±5.0	24.9±4.4	0.3 (−2.8 to 3.4)	0.506
Carbohydrate (%kcal)	54.2±7.1	52.9±8.4	−1.3 (−5.3 to 2.8)	53.9±5.5	55.6±4.8	1.7 (−2.3 to 5.7)	0.272
Potassium (mg/day)	2734±748	2377±529	−357 (−575 to −138)	2948±698	2737±523	−211 (−511 to 90)	0.403
Calcium (mg/day)	537±173	475±166	−62 (−141 to 17)	551±142	541±136	−10 (−67 to 47)	0.255
Magnesium (mg/day)	288±85	243±60	−45 (−73 to −17)	295±46	271±43	−24 (−43 to −4.6)	0.201
Phosphorus (mg/day)	1074±207	980±212	−94 (−187 to −1)	1113±189	1015±108	−98 (−189 to −8.1)	0.941
Iron (mg/day)	8.1±2.1	7.0±1.6	−1.2 (−2.0 to −0.3)	8.7±1.2	7.5±1.4	−1.1 (−2.0 to −0.3)	0.988
α-Tocopherol (mg/day)	6.9±1.2	6.5±1.6	−0.4 (−1.2 to 0.5)	7.8±1.9	7.0±1.8	−0.8 (−2.1 to −0.6)	0.575
Vitamin B_1_ (mg/day)	0.90±0.18	0.80±0.24	−0.10 (−0.22 to 0.02)	0.96±0.21	0.89±0.21	−0.07 (−0.22 to 0.07)	0.772
Total dietary fiber (g/day)	13.9±4.5	19.5±3.2	5.6 (3.9 to 7.3)	16.5±3.1	15.3±3.0	−1.2 (−3.1 to 0.8)	<0.0001
Insoluble dietary fiber (g/day)	9.9±3.1	14.1±2.7	4.1 (2.9 to 5.4)	12.2±2.5	11.3±2.5	−0.9 (−2.5 to 0.8)	<0.0001
Soluble dietary fiber (g/day)	3.1±1.1	4.6±0.8	1.6 (1.1 to 2.1)	3.7±0.8	3.1±0.7	−0.5 (−0.9 to −0.1)	<0.0001
NaCl (g/day)	9.5±2.0	10.0±3.1	0.4 (−1.0 to 1.9)	10.6±1.3	9.6±2.1	−0.9 (−2.0 to 0.2)	0.116

Values are mean ± SD for continuous variables of “baseline” and “during the intervention”, and mean (95% CI) for continuous variables of “Change”. The p-values indicate comparisons of changes between the brown rice diet group *vs*. the white rice diet group using unpaired *t*-tests.

### Changes in metabolic variables after dietary intervention

Changes in metabolic variables during the study period are shown in Table 3. No differences in bodyweight, body fat, and blood pressure were observed between the two groups. Fasting plasma glucose levels decreased at T1 in the brown rice diet group, but the between-group differences were not statistically significant (−0.44 mmol/L *vs*. −0.06 mmol/L; a difference of −0.38 mmol/L [95% CI for the difference, −1.02–0.25]) (Table 4). HbA_1c_ levels slightly improved in both groups, but surprisingly, there was no statistically significant difference between the two groups (−2.6 mmol/mol *vs*. −1.2 mmol/mol; a difference of −1.3 mmol/mol [95% CI for the difference, −4.5–1.8]). Total and LDL-cholesterol levels decreased at T1 in the brown rice diet group, but there were no between-group differences (−0.14 mmol/L *vs*. −0.01 mmol/L; a difference of −0.13 mmol/L [95% CI for the difference, −0.5–0.2]). To analyze the role of systemic inflammation, we measured hs-CRP levels; these levels tended to improve at T1 in the brown rice diet group compared with the white rice diet group (−0.04 μg/L *vs*. 0.01 μg/L; a difference of −0.05 μg/L [95% CI for the difference, −0.10–0.00]). There were no between-group differences in changes of fasting insulin, HOMA-IR, HOMA-β, the oxidative stress marker urine 8-isoprostane, tPAI-1, ADMA, and adiponectin.

**Table 3:** Changes in metabolic variables in the brown rice and white rice diet groups.

	Brown rice group			White rice group			P value
	Baseline (T0)	The end of intervention (T1)	Change	Baseline (T0)	The end of intervention (T1)	Change	
Body weight (kg)	63.2±9.9	62.6±9.7	−0.6 (−1.3 to 0.1)	66.3±14.0	66.0±14.2	−0.3 (−0.7 to 0.1)	0.468
Body mass index (kg/m^2^)	24.2±3.5	24.0±3.4	−0.2 (−0.5 to 0.0)	25.0±3.7	24.9±3.7	−0.1 (−0.3 to 0.0)	0.463
Body fat (%)	26.2±6.9	26.4±7.9	0.2 (−2.0 to 2.4)	28.6±9.2	28.3±9.4	−0.4 (−1.6 to 0.9)	0.647
SBP (mmHg)	121.9±15.0	119.0±12.4	−2.9 (−9.7 to 3.8)	124.9±15.9	120.1±12.5	−4.8 (−12.2 to 2.6)	0.687
DBP (mmHg)	70.7±8.2	68.4±8.3	−2.3 (−5.8 to 1.2)	69.1±9.7	69.0±7.8	−0.1 (−3.3 to 3.0)	0.336
Fasting plasma glucose (mmol/L)	6.79±1.02	6.35±0.86	−0.44 (−0.98 to 0.10)	7.04±1.30	6.98±1.54	−0.06 (−0.46 to 0.34)	0.228
Fasting plasma insulin (pmol/L)	35.4±37.2	35.0±45.7	-0.39 (-7.44 to 6.66)	38.2±20.2	41.6±28.1	3.42 (-8.06 to 14.89)	0.547
HbA1c (%)	6.7±0.6	6.5±0.4	−0.2 (−0.5 to 0.0)	6.8±0.6	6.7±0.6	−0.1 (−0.3 to 0.1)	0.391
(mmol/mol)	50.0±6.5	47.5±4.6	−2.6 (−5.0 to −0.1)	51.1±7.0	49.8±6.2	−1.2 (−3.5 to 1.0)	0.391
Glycoalbumin (%)	17.0±2.5	16.7±2.1	−0.4 (−1.2 to 0.5)	18.0±2.8	18.2±2.7	0.2 (−0.6 to 1.0)	0.330
HOMA-IR	1.92±2.42	1.83±3.02	−0.08 (−0.61 to 0.44)	2.02±1.24	2.09±1.34	0.07 (−0.47 to 0.61)	0.665
HOMA-β	35.6±29.2	38.8±33.7	3.2 (-6.4 to 12.8)	41.7±30.3	56.3±68.8	14.6 (-13.8 to 43)	0.423
Total cholesterol (mmol/L)	5.10±0.96	4.89±0.81	−0.21 (−0.51 to 0.09)	4.88±0.85	4.90±0.78	0.01 (−0.24 to 0.27)	0.235
HDL cholesterol (mmol/L)	1.65±0.42	1.54±0.35	−0.11 (−0.19 to −0.04)	1.41±0.29	1.40±0.31	−0.01 (−0.12 to 0.10)	0.100
LDL cholesterol (mmol/L)	3.05±0.63	2.91±0.67	−0.14 (−0.40 to 0.13)	2.98±0.65	2.98±0.62	−0.01 (−0.23 to 0.21)	0.431
Triglyceride (mmol/L)	465±155	514±161	48.9 (−18.0 to 115.7)	562±523	597±650	35.6 (−49.3 to 120.4)	0.793
High sensitivity CRP (μg/L)	0.09±0.12	0.05±0.05	−0.04 (−0.09 to 0.01)	0.04±0.03	0.05±0.06	0.01 (−0.01 to 0.03)	0.063
tPAI-1 (ng/mL)	17.6±13.6	15.5±10.5	−2.1 (−7.2 to 3.1)	16.3±7.2	16.2±6.0	−0.1 (−2.5 to 2.4)	0.458
Adiponectin (ng/mL)	8.2±4.6	7.7±4.3	−0.5 (−1.2 to 0.2)	7.9±4.6	8.1±5.0	0.2 (−0.2 to 0.7)	0.057
ADMA (μmol/L)	0.50±0.12	0.43±0.12	−0.08 (−0.15 to 0.00)	0.52±0.18	0.50±0.16	−0.02 (−0.13 to 0.09)	0.404
Urine 8-isoprostane (pg/mg·cre)	152.3±67.3	161.2±45.2	8.9 (−26.8 to 44.5)	150.9±67.8	158.9±68.5	8.0 (−18.9 to 34.9)	0.968

Values are mean ± SD for continuous variables of “T0” and “T1”, and mean (95% CI) for continuous variables of “Change”. The p-values indicate comparisons of changes between the brown rice diet group *vs*. the white rice diet group using unpaired *t*-tests. HOMA, homeostasis model assessment; LDL, low-density lipoprotein; HDL, high-density lipoprotein; tPAI-1, tissue plasminogen activator-1; hs-CRP, high-sensitivity C-reactive protein; HbA1c, glycated hemoglobin; ADMA, asymmetric dimethylarginine.

**Table 4:** Changes in endothelial function in the brown rice and white rice diet groups.

	Brown rice group				White rice group				P value between randomized groups
	Baseline (T0)	The end of intervention (T1)	Change	P value	Baseline (T0)	The end of intervention (T1)	Change	P value	
Fasting FDR (%)	46.3±17.1	66.7±34.5	20.4 (5.1 to 35.7)	0.013	47.5±23.2	41.7±19.7	-5.8 (-13.7 to 2.1)	0.136	0.004
Fasting Peak FBF (%)	407.7±130.1	526.6±199.9	118.8 (40.4 to 197.2)	0.006	387.1±192.4	343.8±157.9	-43.3 (-109.5 to 22.9)	0.179	0.002
Fasting duration of RH (sec)	59.6±20.5	69.3±23.9	9.7 (1.9 to 17.6)	0.019	62.1±14.2	61±12.5	-1.1 (-3.1 to 0.9)	0.266	0.012
Postprandial FDR (%)	31.3±9	49.2±23.2	17.9 (3.8 to 32)	0.017	38.6±19.5	34.2±19.2	-4.4 (-16.3 to 7.5)	0.435	0.015
Postprandial Peak FBF (%)	329.9±71	437±189.5	107.2 (2 to 212.3)	0.046	342.9±172.9	290.3±152.3	-52.6 (-134.8 to 29.6)	0.189	0.017
Postprandial duration of RH (sec)	50.1±15.4	61.9±15	11.8 (2.7 to 20.9)	0.015	51.4±15.1	52.4±9.3	1 (-5.5 to 7.5)	0.744	0.050

Values are mean ± SD for continuous variables of “T0” and “T1”, and mean (95% CI) for continuous variables of “Change”. The p-values indicate comparisons of changes between the brown rice diet group *vs*. the white rice diet group using unpaired *t*-tests. FDR, flow debt repayment; FBF, forearm blood flow; RH, reactive hyperemia.

### Rice tolerance test before and after the intervention

**Fig 3:**
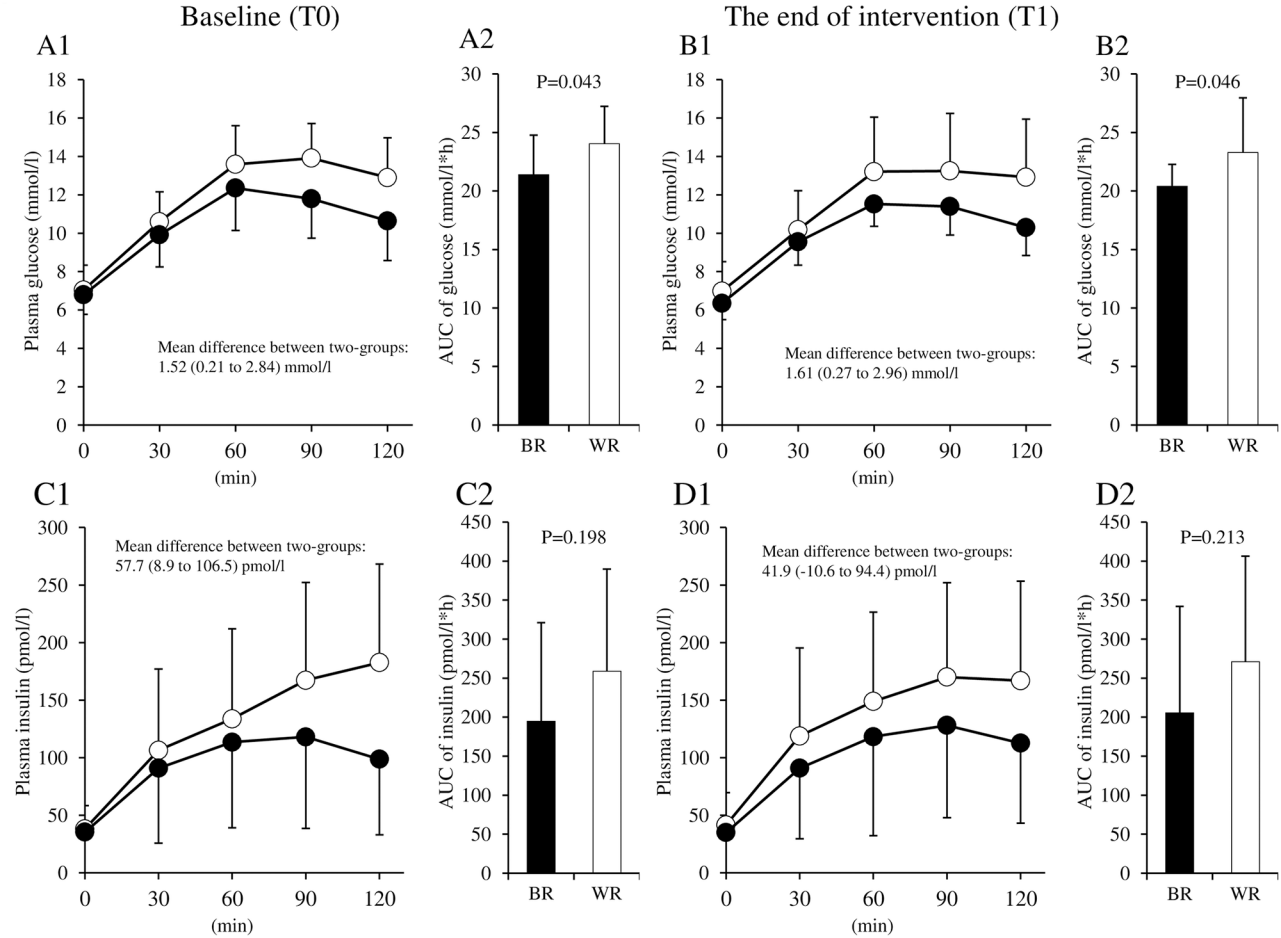
Changes in plasma glucose and insulin levels during the rice tolerance test. Levels of plasma glucose (A1, B1) and the area under the curve (AUC) for plasma glucose (A2, B2) during the rice tolerance test at baseline (T0: left graph) and at the end of intervention (T1: right graph) for estimating the postprandial state during the intervention. Levels of plasma insulin and AUC were measured during the rice tolerance test (C1–2, D1–2). BR, brown rice diet group (black circle and box), WR, white rice diet group (white circle and box).

Acute and chronic effects of brown rice on glucose excursions were estimated using rice tolerance tests: a brown rice tolerance test for the brown rice diet group and a white rice tolerance test for the white rice diet group. Rice tolerance tests showed lower postprandial glucose increments in the brown rice diet group compared with the white rice diet group at T0 (mean difference between groups: 1.52 mmol/L [95% CI, 0.21–2.84], AUC: 21.4 mmol/L/h *vs*. 24.0 mmol/L/h, p = 0.043) (Fig 3). Rice tolerance tests at T1 also showed that increments in plasma glucose concentrations were lower in the brown rice diet group compared with the white rice diet group (mean difference between groups: 1.61 mmol/L [95% CI, 0.27–2.96], AUC: 20.4 mmol/L/h *vs*. 23.3 mmol/L/h, p = 0.046), with levels within groups similar at T0 and T1. These results suggested that glucose excursions were consistent throughout the intervention in both the brown and white rice diet groups.

The effect of brown rice on postprandial insulin levels was also estimated using rice tolerance tests. Rice tolerance tests showed lower postprandial insulin increments in the brown rice diet group compared with the white rice diet group at T0 (mean difference between groups: 57.7 pmol/L [95% CI, 8.9–106.5], AUC: 194.8 pmol/L*h *vs*. 259.0 pmol/L*h, p = 0.198) (Fig 3). Rice tolerance tests at T1 also showed that increments in plasma insulin concentrations were lower in the brown rice diet group compared with the white rice diet group (mean difference between groups: 41.9 pmol/L [95% CI, −10.6–94.4], AUC: 205.5 pmol/L*h *vs*. 271.0 pmol/L*h, p = 0.213), with levels within groups similar at T0 and T1.

### Changes in endothelial function after dietary intervention

Changes in endothelial function, evaluated by strain-gauge plethysmography, for each group are shown in Table 4. There was greater improvement of fasting FDR (primary outcome) following 8 weeks of intervention in the brown rice diet group compared with the white rice diet group (20.4% *vs*. −5.8%; a difference of 26.2% [95% CI for the difference, 9.4–42.8], p = 0.002 using the Wilcoxon test). Adjusted analysis showed consistency in the effects of the brown rice diet compared with the white rice diet on fasting FDR (20.1% *vs*. −5.5%; a difference of 25.7% [95% CI for the difference, 1.2–50.1]). The improvements in fasting peak FBF and duration of RH were also greater in the brown rice diet group compared with the white rice diet group (peak FBF: 118.8% *vs*. −43.3%; a difference of 162.1% [95% CI for the difference, 63.9–260.4], duration of RH: 9.7 s *vs*. −1.1 s; a difference of 10.8 s [95% CI for the difference, 2.8–18.8]).

There were also greater improvements in FDR, peak FBF, and duration of RH in the postprandial state in the brown rice diet group compared with the white rice diet group (FDR: 17.9% *vs*. −4.4%; a difference of 22.3% [95% CI for the difference, 4.7–39.9], peak FBF: 107.2% *vs*. −52.6%; a difference of 159.8 [95% CI for the difference, 31.6–288.0], duration of RH: 11.8 s *vs*. 1 s; a difference of 10.8 s [95% CI for the difference, 0–21.6]).

## Discussion

This study demonstrated two major findings. First, intervention with a fiber-rich diet with brown rice improved endothelial function in patients with T2DM without changes in bodyweight. Second, rice tolerance tests at both baseline (T0) and at the end of intervention (T1) showed that glucose excursions were lower in the brown rice diet group than in the white rice diet group, and that this difference remained at the end of the intervention.

Intervention with a fiber-rich diet of brown rice improved endothelial function in patients with T2DM. These findings are in agreement with results from a previous study that reported improved endothelial function with brown rice consumption in participants with metabolic syndrome [21]. However, compared with the previous study, our study differs in terms of the background of participants, weight reduction, and the methods for evaluating endothelial function (flow-mediated vasodilation). To rule out the effects of bodyweight reduction, we carefully controlled participants’ bodyweights to prevent a difference between the two groups during the study. To the best of our knowledge, our study is the first to report that a fiber-rich diet with brown rice improves endothelial function in patients with T2DM. We believe that this phenomenon may contribute to the cardioprotective effects of dietary fiber.

As well as improved endothelial function, glucose excursions estimated with AUC for glucose using rice tolerance tests were lower in the brown rice diet group at both T0 and T1 compared with the white rice diet group. This finding aligns with a previous study that reported that dietary fiber reduced plasma glucose concentrations after a meal [22]. This glucose spike is a proposed mechanism of both microvascular and macrovascular diabetic complications [23]. In the current study, we observed no significant difference in HbA_1c_ levels between the two groups, suggesting that a subtle but cumulative reduction in plasma glucose concentrations might explain the improvements in endothelial function without changes in HbA_1c_ levels and oxidative stress markers.

Inflammation may be a key factor connecting endothelial function and a high-fiber diet. It has been reported that the intake of dietary fiber is inversely associated with serum hs-CRP levels [24]. In addition, increased dietary fiber intake from a diet naturally rich in fiber and supplements can reduce hs-CRP levels [14]. We previously reported that improvements in inflammatory markers after a high-fiber, low-fat diet intervention might be related to both the composition of the diet and bodyweight reduction [25]. In the current study, hs-CRP levels tended to be reduced at T1 in the brown rice diet group compared with the white rice diet group, but not significantly.

Hyperinsulinemia is a cause of endothelial dysfunction [26]. In our study, postprandial insulin levels, estimated by rice tolerance tests, were relatively lower in the brown rice group compared with the white rice group (Fig 3). This might also partially explain improved endothelial function at T1 in the brown rice diet group. Dyslipidemia is one of the mechanisms underlying endothelial dysfunction [27]. Dietary fiber can reduce total and LDL-cholesterol levels in patients with hypercholesterolemia or T2DM [28]. However, we did not see significant differences in serum levels of total, HDL-, and LDL-cholesterol and triglyceride between the two groups. Triglyceride levels after the rice tolerance test showed no differences in both groups (data not shown). Levels of adiponectin tended to be reduced after the intervention in the brown rice diet group compared with the white rice diet group with and without BMI adjustment. The mechanism underlying this phenomenon is unknown, but it seems that the magnitude of the change was too subtle to affect endothelial function in participants in the current study. Serum ADMA and urine 8-isoprostane levels showed no significant differences between the two groups. These findings suggest that these effects of a fiber-rich diet might not be major causes of the observed improvement in endothelial function.

Brown rice contains high levels of fiber compared with white rice. Accumulated evidence suggests that dietary composition is one of the determinants of the microflora [29]. Although the relationships between atherosclerosis and endothelial function with microflora remain unclear, consideration of the contribution of the microflora paradigm—as in the case of trimethylamine-N-oxide in cardiovascular events—may open a new research window in this area [30]. Brown rice contains high levels of vitamins and minerals, particularly vitamin B_1_ and magnesium [16]. These nutrients may be associated with the observed improvements in endothelial function. Supplementation of magnesium has been reported to attenuate insulin resistance in patients with T2DM [31]. However, in the current study, levels of these nutrients were unchanged during the intervention (T1) in both groups. This suggests that dietary fiber, rather than these micronutrients, could explain the improvement in endothelial function with brown rice intake.

The strengths of this study are two-fold. First, a relatively mild increase in fiber intake improved endothelial function. The dietary intervention in this study appears to be feasible for clinical practice, at least in a Japanese population. Second, the intervention was not associated with changes in HbA_1c_ levels although rice tolerance tests showed significantly lower plasma glucose concentrations with a brown rice diet compared with a white rice diet. Along with previous interventions, findings from the current study may support the “glucose spike” theory, because a small difference between groups was associated with a large difference in endothelial function.

This study has some limitations. First, the number of participants was relatively small, although a sufficient sample size was calculated. Therefore, baseline characteristics between the two groups might be imbalanced. Second, glucose excursions were estimated by rice tolerance tests with assigned rice types. We used the rice tolerance test to determine glucose excursions in each group; assessment with continuous glucose monitoring systems would be more favorable. Because each group consumed a different type of rice for the rice tolerance tests, caution is necessary when comparing the results for direct comparisons between the two groups as a randomized controlled trial. Third, the presence of prior cardiovascular events was not an exclusion criterion. This factor might affect endothelial function in these individuals. Fourth, real increases in fiber intake in the brown rice diet group are unclear because no information on dietary fiber intake before the run-in period was recorded. Fifth, we used plethysmography to evaluate endothelial function in this study. Although this is a validated method, caution needs to be taken when comparing the results of the current study with studies that used other methods, such as EndoPAT and flow-mediated vasodilation. Finally, the study participants were recruited at a single hospital. Therefore, any generalizations must be made with caution.

In conclusion, intervention with a fiber-rich diet with brown rice for 8 weeks effectively improved endothelial function, without changing HbA_1c_ levels, possibly through reducing glucose excursions.

## Supporting information

S1 Fig Schematic representation of a reactive hyperemic response and the index calculation. Forearm blood flow (FBF) was measured with a mercury-filled Silastic (Dow Corning, Midland, MI, USA) strain-gauge plethysmograph (EC-6; D.E. Hokanson, Bellevue, WA, USA) using a venous occlusion technique. The increase in forearm volume was measured after blocking the venous efflux by an upper arm cuff inflated to 50 mmHg by a rapid cuff inflator (Hokanson E20, Bellevue, WA, USA) for 7 s during each 15 s cycle to determine FBF. FBF (mL·100 mL forearm^-1^·min^-1^) was calculated using specialized software (Noninvasive Vascular Program 3 (NIVP3), Hokanson, Bellevue, WA, USA) which calculated the slope from the change in forearm volume over time and determined blood flow as percent volume change per minute (%·min^-1^). To produce reactive hyperemia, blood flow to the forearm was prevented by inflation of the cuff on the right upper arm to a pressure of 190 mmHg when systolic blood pressure (SBP) was ≤140 mmHg, or 50 mmHg plus SBP when SBP was >140 mmHg. The duration of arterial occlusion was 5 min. After release of arterial occlusion, FBF was measured at 7 s after release and every 15 s thereafter. FBF were monitored and recorded continuously during reactive hyperemia. Three variables were evaluated for endothelial function: 1) Peak FBF (%) = (b / a) × 100; 2) Duration (s) = c distance; and 3) Flow debt repayment (FDR) (%) = (β area / α area) × 100. a: Forearm blood flow (mL·100 mL forearm^-1^·min^-1^) at rest, b: FBF at peak value after release of occlusion, c: Duration of hyperemia after release of occlusion, α: The area under the curve between the start and the end of the occlusion period, β: The area under the curve between the release of occlusion and the duration of hyperemia.
(TIF)

S2 Fig Changes in fasting flow debt repayment (FDR) (primary outcome) in each participant. Data are shown as raw data (A, B) and the change from T0 to T1 (C, D). Black circle: brown rice group (A, C). White circle: white rice group (B, D).
(TIF)

S1 Table Nutritional compositions of both brown rice and white rice. (DOCX)

S1 File CONSORT checklist. (DOCX)

S2 File Original protocol in Japanese. (DOC)

S3 File Original protocol in English. (DOC)

S4 File Final protocol in Japanese. (DOC)

S5 File Final protocol in English. (DOC)

## Abbreviations

ADMA: asymmetric dimethylarginine
AUC: area under the curve
BMI: body mass index
FBF: forearm blood flow
FDR: flow debt repayment
HbA1c: glycated hemoglobin
HDL: high-density lipoprotein
Hs-CRP: high-sensitivity C-reactive protein
LDL: low-density lipoprotein
RH: reactive hyperemia
SBP: systolic blood pressure
T2DM: type 2 diabetes mellitus
tPAI-1: tissue plasminogen activator inhibitor-1

## References

[1] Wolk A, Manson JE, Stampfer MJ, Colditz GA, Hu FB, Speizer FE, et al Long-term intake of dietary fiber and decreased risk of coronary heart disease among women. Jama. 1999;281(21):1998–2004. Epub 1999/06/08. . 10359388 10.1001/jama.281.21.1998

[2] Park Y, Subar AF, Hollenbeck A, Schatzkin A. Dietary fiber intake and mortality in the NIH-AARP diet and health study. Archives of internal medicine. 2011;171(12):1061–8. Epub 2011/02/16. doi: 10.1001/archinternmed.2011.18 ; 21321288 10.1001/archinternmed.2011.18 PMC3513325

[3] Estruch R, Ros E, Salas-Salvado J, Covas MI, Corella D, Aros F, et al Primary prevention of cardiovascular disease with a Mediterranean diet. N Engl J Med. 2013;368(14):1279–90. doi: 10.1056/NEJMoa1200303 . 23432189 10.1056/NEJMoa1200303

[4] Perticone F, Ceravolo R, Pujia A, Ventura G, Iacopino S, Scozzafava A, et al Prognostic significance of endothelial dysfunction in hypertensive patients. Circulation. 2001;104(2):191–6. Epub 2001/07/12. . 11447085 10.1161/01.cir.104.2.191

[5] Yeboah J, Crouse JR, Hsu FC, Burke GL, Herrington DM. Brachial flow-mediated dilation predicts incident cardiovascular events in older adults: the Cardiovascular Health Study. Circulation. 2007;115(18):2390–7. doi: 10.1161/CIRCULATIONAHA.106.678276 . 17452608 10.1161/CIRCULATIONAHA.106.678276

[6] Hamilton SJ, Chew GT, Watts GF. Therapeutic regulation of endothelial dysfunction in type 2 diabetes mellitus. Diab Vasc Dis Res. 2007;4(2):89–102. Epub 2007/07/27. doi: 10.3132/dvdr.2007.026 . 17654442 10.3132/dvdr.2007.026

[7] Rallidis LS, Lekakis J, Kolomvotsou A, Zampelas A, Vamvakou G, Efstathiou S, et al Close adherence to a Mediterranean diet improves endothelial function in subjects with abdominal obesity. The American journal of clinical nutrition. 2009;90(2):263–8. Epub 2009/06/12. doi: 10.3945/ajcn.2008.27290 . 19515732 10.3945/ajcn.2008.27290

[8] Kondo K, Morino K, Nishio Y, Kondo M, Nakao K, Nakagawa F, et al A fish-based diet intervention improves endothelial function in postmenopausal women with type 2 diabetes mellitus: a randomized crossover trial. Metabolism. 2014;63(7):930–40. Epub 2014/05/23. doi: 10.1016/j.metabol.2014.04.005 . 24850465 10.1016/j.metabol.2014.04.005

[9] Vita JA. Endothelial function. Circulation. 2011;124(25):e906–12. doi: 10.1161/CIRCULATIONAHA.111.078824 . 22184047 10.1161/CIRCULATIONAHA.111.078824

[10] McGill CR, Fulgoni VL 3rd, Devareddy L. Ten-year trends in fiber and whole grain intakes and food sources for the United States population: National Health and Nutrition Examination Survey 2001–2010. Nutrients. 2015;7(2):1119–30. Epub 2015/02/12. doi: 10.3390/nu7021119 ; 25671414 10.3390/nu7021119 PMC4344579

[11] Ministry of Health and Welfare. Annual report of the National Nutrition Survey in 2011: Daiichi Publishing; 2015.

[12] Evert AB, Boucher JL, Cypress M, Dunbar SA, Franz MJ, Mayer-Davis EJ, et al Nutrition therapy recommendations for the management of adults with diabetes. Diabetes Care. 2014;37 Suppl 1:S120–43. Epub 2013/12/21. doi: 10.2337/dc14-S120 . 24357208 10.2337/dc14-S120

[13] Yao B, Fang H, Xu W, Yan Y, Xu H, Liu Y, et al Dietary fiber intake and risk of type 2 diabetes: a dose-response analysis of prospective studies. Eur J Epidemiol. 2014;29(2):79–88. Epub 2014/01/07. doi: 10.1007/s10654-013-9876-x . 24389767 10.1007/s10654-013-9876-x

[14] King DE, Egan BM, Woolson RF, Mainous AG 3rd, Al-Solaiman Y, Jesri A. Effect of a high-fiber diet vs a fiber-supplemented diet on C-reactive protein level. Archives of internal medicine. 2007;167(5):502–6. Epub 2007/03/14. doi: 10.1001/archinte.167.5.502 . 17353499 10.1001/archinte.167.5.502

[15] Qi L, Meigs JB, Liu S, Manson JE, Mantzoros C, Hu FB. Dietary fibers and glycemic load, obesity, and plasma adiponectin levels in women with type 2 diabetes. Diabetes Care. 2006;29(7):1501–5. Epub 2006/06/28. doi: 10.2337/dc06-0221 . 16801569 10.2337/dc06-0221

[16] Resources Council, Science and Technology Agency. standard tables of food composition in Japan. Tokyo: National printing bureau; 2010.

[17] The Japan Diabetes Society. Treatment Guide for Diabetes 2013, http://www.jds.or.jp/modules/publication/index.php?content_id=4.

[18] Linder L, Kiowski W, Buhler FR, Luscher TF. Indirect evidence for release of endothelium-derived relaxing factor in human forearm circulation in vivo. Blunted response in essential hypertension. Circulation. 1990;81(6):1762–7. Epub 1990/06/01. . 2344673 10.1161/01.cir.81.6.1762

[19] Higashi Y, Sasaki S, Nakagawa K, Matsuura H, Kajiyama G, Oshima T. A noninvasive measurement of reactive hyperemia that can be used to assess resistance artery endothelial function in humans. Am J Cardiol. 2001;87(1):121–5, a9 Epub 2001/01/04. . 11137850 10.1016/s0002-9149(00)01288-1

[20] Tagawa T, Imaizumi T, Endo T, Shiramoto M, Harasawa Y, Takeshita A. Role of nitric oxide in reactive hyperemia in human forearm vessels. Circulation. 1994;90(5):2285–90. Epub 1994/11/01. . 7955185 10.1161/01.cir.90.5.2285

[21] Shimabukuro M, Higa M, Kinjo R, Yamakawa K, Tanaka H, Kozuka C, et al Effects of the brown rice diet on visceral obesity and endothelial function: the BRAVO study. Br J Nutr. 2014;111(2):310–20. Epub 2013/08/13. doi: 10.1017/S0007114513002432 . 23930929 10.1017/S0007114513002432

[22] Marangoni F, Poli A. The glycemic index of bread and biscuits is markedly reduced by the addition of a proprietary fiber mixture to the ingredients. Nutr Metab Cardiovasc Dis. 2008;18(9):602–5. Epub 2008/04/05. doi: 10.1016/j.numecd.2007.11.003 . 18387793 10.1016/j.numecd.2007.11.003

[23] Gerich JE. Clinical significance, pathogenesis, and management of postprandial hyperglycemia. Archives of internal medicine. 2003;163(11):1306–16. Epub 2003/06/11. doi: 10.1001/archinte.163.11.1306 . 12796066 10.1001/archinte.163.11.1306

[24] Ajani UA, Ford ES, Mokdad AH. Dietary fiber and C-reactive protein: findings from national health and nutrition examination survey data. J Nutr. 2004;134(5):1181–5. Epub 2004/04/29. . 15113967 10.1093/jn/134.5.1181

[25] Kondo K, Ishikado A, Morino K, Nishio Y, Ugi S, Kajiwara S, et al A high-fiber, low-fat diet improves periodontal disease markers in high-risk subjects: a pilot study. Nutr Res. 2014;34(6):491–8. Epub 2014/07/17. doi: 10.1016/j.nutres.2014.06.001 . 25026916 10.1016/j.nutres.2014.06.001

[26] Arcaro G, Cretti A, Balzano S, Lechi A, Muggeo M, Bonora E, et al Insulin causes endothelial dysfunction in humans: sites and mechanisms. Circulation. 2002;105(5):576–82. Epub 2002/02/06. . 11827922 10.1161/hc0502.103333

[27] Creager MA, Cooke JP, Mendelsohn ME, Gallagher SJ, Coleman SM, Loscalzo J, et al Impaired vasodilation of forearm resistance vessels in hypercholesterolemic humans. The Journal of clinical investigation. 1990;86(1):228–34. Epub 1990/07/01. doi: 10.1172/JCI114688 ; 2195060 10.1172/JCI114688 PMC296711

[28] Whitehead A, Beck EJ, Tosh S, Wolever TM. Cholesterol-lowering effects of oat beta-glucan: a meta-analysis of randomized controlled trials. The American journal of clinical nutrition. 2014;100(6):1413–21. Epub 2014/11/21. doi: 10.3945/ajcn.114.086108 . 25411276 10.3945/ajcn.114.086108 PMC5394769

[29] Canfora EE, Jocken JW, Blaak EE. Short-chain fatty acids in control of body weight and insulin sensitivity. Nat Rev Endocrinol. 2015 Epub 2015/08/12. doi: 10.1038/nrendo.2015.128 . 26260141 10.1038/nrendo.2015.128

[30] Tang WH, Wang Z, Levison BS, Koeth RA, Britt EB, Fu X, et al Intestinal microbial metabolism of phosphatidylcholine and cardiovascular risk. N Engl J Med. 2013;368(17):1575–84. Epub 2013/04/26. doi: 10.1056/NEJMoa1109400 ; 23614584 10.1056/NEJMoa1109400 PMC3701945

[31] Rodriguez-Moran M, Guerrero-Romero F. Oral magnesium supplementation improves insulin sensitivity and metabolic control in type 2 diabetic subjects: a randomized double-blind controlled trial. Diabetes Care. 2003;26(4):1147–52. Epub 2003/03/29. . 12663588 10.2337/diacare.26.4.1147

