# Supplementary figures and images for: Fiber-rich diet with brown rice improves endothelial function in type 2 diabetes mellitus: A randomized controlled trial
Source: PLoS One. 2017 Jun 29;12(6):e0179869. doi: 10.1371/journal.pone.0179869 (PMC5491061; doi:10.1371/journal.pone.0179869)

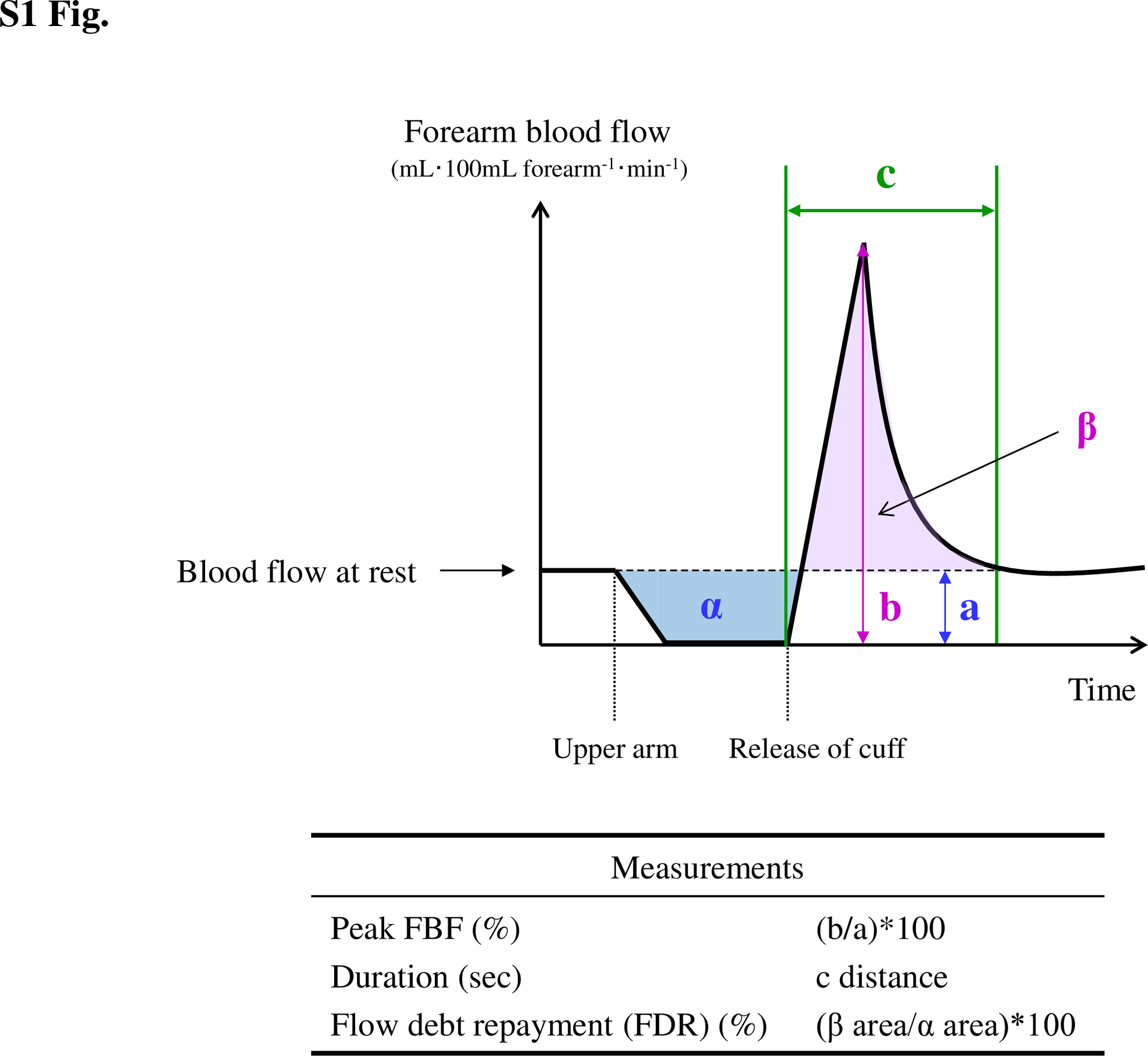

Supplement: S1 Fig — Forearm blood flow (FBF) was measured with a mercury-filled Silastic (Dow Corning, Midland, MI, USA) strain-gauge plethysmograph (EC-6; D.E. Hokanson, Bellevue, WA, USA) using a venous occlusion technique. The increase in forearm volume was measured after blocking the venous efflux by an upper arm cuff inflated to 50 mmHg by a rapid cuff inflator (Hokanson E20, Bellevue, WA, USA) for 7 s during each 15 s cycle to determine FBF. FBF (mL·100 mL forearm-1·min-1) was calculated using specialized software (Noninvasive Vascular Program 3 (NIVP3), Hokanson, Bellevue, WA, USA) which calculated the slope from the change in forearm volume over time and determined blood flow as percent volume change per minute (%·min-1). To produce reactive hyperemia, blood flow to the forearm was prevented by inflation of the cuff on the right upper arm to a pressure of 190 mmHg when systolic blood pressure (SBP) was ≤140 mmHg, or 50 mmHg plus SBP when SBP was >140 mmHg. The duration of arterial occlusion was 5 min. After release of arterial occlusion, FBF was measured at 7 s after release and every 15 s thereafter. FBF were monitored and recorded continuously during reactive hyperemia. Three variables were evaluated for endothelial function: 1) Peak FBF (%) = (b / a) × 100; 2) Duration (s) = c distance; and 3) Flow debt repayment (FDR) (%) = (β area / α area) × 100. a: Forearm blood flow (mL·100 mL forearm-1·min-1) at rest, b: FBF at peak value after release of occlusion, c: Duration of hyperemia after release of occlusion, α: The area under the curve between the start and the end of the occlusion period, β: The area under the curve between the release of occlusion and the duration of hyperemia. (TIF) [file pone.0179869.s001.tif]

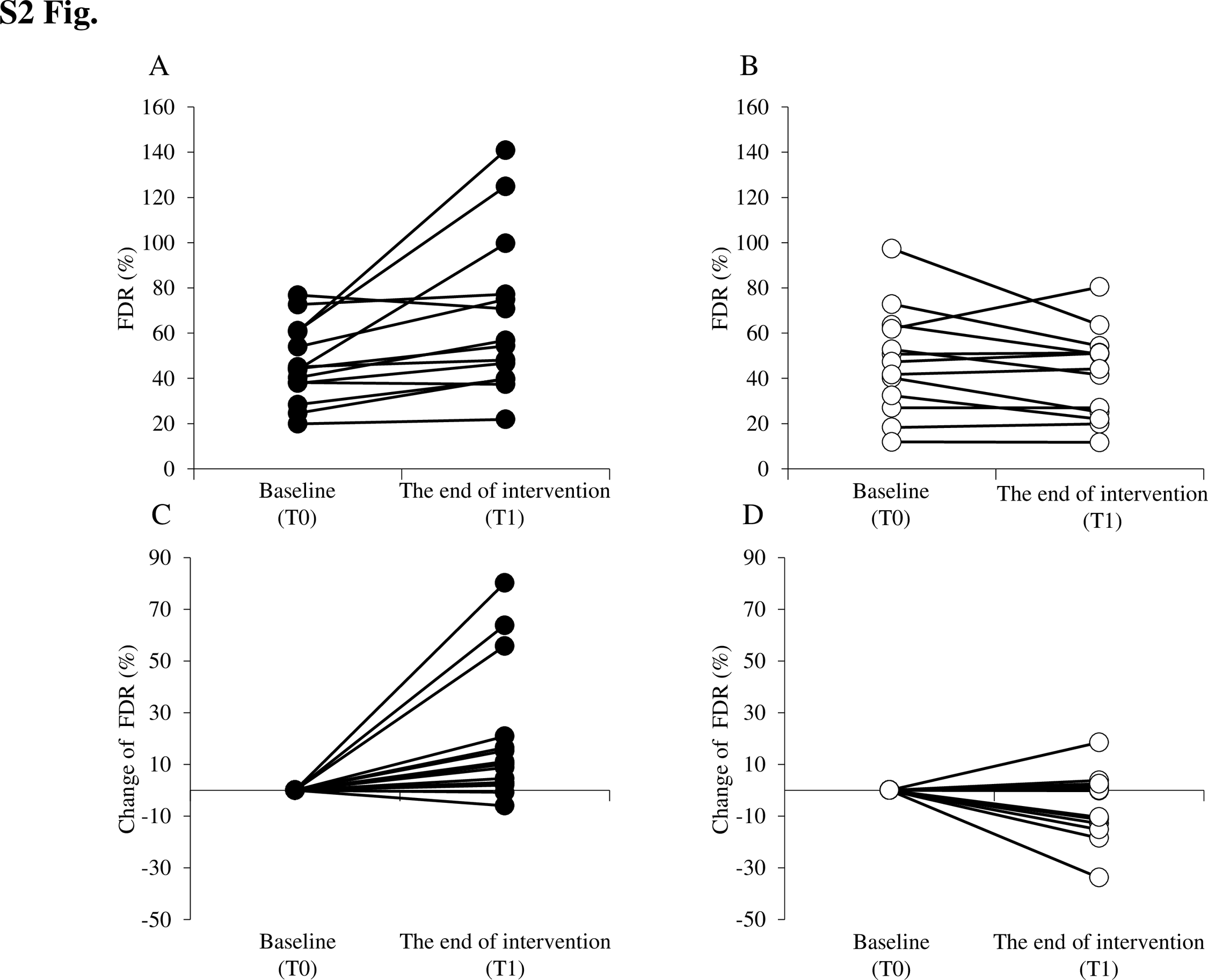

Supplement: S2 Fig — Data are shown as raw data (A, B) and the change from T0 to T1 (C, D). Black circle: brown rice group (A, C). White circle: white rice group (B, D). (TIF) [file pone.0179869.s002.tif]
