# Supplementary material for: Fiber-rich diet with brown rice improves endothelial function in type 2 diabetes mellitus: A randomized controlled trial
Source: PLoS One. 2017 Jun 29;12(6):e0179869. doi: 10.1371/journal.pone.0179869 (PMC5491061; doi:10.1371/journal.pone.0179869)
Supplement: S1 Table — (DOCX) [file pone.0179869.s003.docx]

**S1 Table. Nutritional compositions of both brown rice and white rice.**

|  | Brown rice | White rice |
| --- | --- | --- |
| Energy (kcal) | 250 | 250 |
| Weight (g) | 182 | 153 |
| Protein (g) | 5.0 | 4.2 |
| Fat (g) | 2.5 | 0.6 |
| Carbohydrate (g) | 54.3 | 57.3 |
| Total dietary fiber (g) | 4.3 | 0.5 |
| Insoluble fiber (g) | 1.2 | 0.0 |
| Soluble fiber (g) | 3.1 | 0.5 |
