# Supplementary material for: Fiber-rich diet with brown rice improves endothelial function in type 2 diabetes mellitus: A randomized controlled trial
Source: PLoS One. 2017 Jun 29;12(6):e0179869. doi: 10.1371/journal.pone.0179869 (PMC5491061; doi:10.1371/journal.pone.0179869)
Supplement: S2 File — (DOC) [file pone.0179869.s005.doc]

**研究実施計画書**

**玄米食および白米食が糖・脂質代謝および血管機能に及ぼす影響**

**１　背景**

我々は、2型糖尿病患者において玄米菜食メニューを基本としたレトルト試験食（玄米ごはん・レトルト惣菜）を用いた食事介入により血糖コントロールおよび血管拡張機能を改善することを報告した。本研究は、より日常生活に即した食事療法を提案するため、主食のみを玄米あるいは白米に置き換えることが、糖・脂質代謝および血管機能などの指標に対する影響について検討する。

**２　目的**

2型糖尿病患者に対して、主食を玄米あるいは白米とする栄養指導を行い糖・脂質代謝および血管機能へ及ぼす影響について検討する。

**３　試験方法**

**１）対象**

滋賀医科大学医学部附属病院に通院中の2型糖尿病患者

選択基準：

①40歳以上80歳未満の患者

②HbA1c＜8.0％の患者

③インスリン治療していない患者

④α-グルコシダーゼ阻害薬を服用していない患者

⑤非喫煙者

除外の基準：

①重症の血管病患者、重症の肝・腎障害患者、癌患者、感染症患者

②試験開始の直前（2ヶ月以内）に薬剤を変更した者

③急激な体重変化(±10%)を起こした者

④1ヶ月以内に他の臨床試験に参加した者

⑤普段の食事で玄米を摂取している者

⑥食物繊維サプリメントを摂取している者

⑦妊婦、授乳婦、その他担当医師が不適切と判断した者

（現在使用している薬剤は基本的に継続するが、過去1ヶ月以内に抗炎症薬・ワーファリンを使用している場合には除外する。）

**２）目標症例数**

目標症例数　30名

**３）試験食**

玄米ごはんおよび白米ごはん

**４）摂取方法**

試験食品を宅配し、玄米ごはんあるいは白米ごはんを2回/日、5日/週摂取する。摂取エネルギーは、現行の栄養指導に従い、主食を試験食品へ置き換える。個人毎の摂取エネルギーの管理は、提供する主食の量およびその他の食事で調整する。実施期間は2ヶ月間とする。

**５）試験デザインおよび試験期間 (図1)**

本試験のデザインは、ランダム化比較試験（RCT：Randomized Controlled Trial）とし、被験者を玄米群または白米群に無作為に割り当てる。各試験期間は以下の通りである。

①エネルギー調整期間 (試験開始前1～2ヶ月間)

試験開始前にエネルギー調整期間を設け、通常の栄養指導を行う。

②玄米または白米食期間 (2ヶ月間)

エネルギー調整期間終了後、被験者を玄米群あるいは白米群に無作為に割り当てる。玄米群は玄米ごはん、白米群は白米ごはんを宅配にて提供し、主食を置き換える。摂取回数は1日2回、週に5日間とする。主食量は、各個人の設定エネルギー量により調整する。

③エネルギー調整期間 (2ヶ月間)

玄米または白米食期間後、①の期間と同様、エネルギー調整期間を設ける。なお、主食は提供しない。

④フォローアップ期間 (試験終了後2ヶ月間)

①～③の期間が終了した後、フォローアップ期間を設ける。この期間中の栄養指導は行わず、自由摂取とする。

＊なお、全試験期間中を通して食事内容は糖尿病学会の発行する糖尿病治療ガイドラインに沿ったものとし、摂取エネルギー量は標準体重1kgあたり28～30kcalとする。

図1 試験デザイン

**６）食事調査**

　　試験開始前・玄米または白米食期間中(0～2ヶ月)・エネルギー調整期間中(2～4ヶ月)に3

日間の食事調査（記録・カメラ撮影）を行い、栄養摂取量を評価する。なお、食事調査で用いるカメラは事前に患者に渡す。

**７）食事負荷試験**

食事負荷試験は、0、2、4ヶ月に実施する。玄米群は玄米、白米群は白米負荷試験を行う。負荷量は各々1パック(約150g)（現在、栄養分析中）とする。

**８）併用薬（ビタミン剤等も含む）**

- 試験期間（4ヶ月間）中の治療薬および投与量の変更は、原則として認めない。ただし、試験期間中に空腹時血糖値が110以下となった場合など、医師の判断により投与量の変更も可とし、ケースカードに記入する。
- 試験期間中、健康食品としてのサプリメントなどの摂取を禁止する。

**４　評価項目**

以下の臨床評価項目について各群における0, 2ヶ月の変化および4ヶ月のキャリーオーバー効果について評価する。

**１）主要評価項目**

血管拡張機能

**２）副次評価項目**

1) 身体計測：身長、体重、腹囲、血圧、体脂肪

2) 血液検査：赤血球、白血球、Hb、Ht、血小板数、AST、ALT、γ-GTP、HbA1c、GA、T-cho、HDL-cho、LDL-cho、tPAI-1、高感度CRP、アディポネクチン、レプチン、ADMA

3) 尿検査：8-イソプロスタン、クレアチニン

4) 食事負荷試験：（0, 30, 60, 90, 120分）血糖、血清インスリン、TG、FFA、

　　　　　　　　 （0, 30, 60, 120分）グルカゴン、GLP-1、GIP

（0, 120分）血管拡張機能

5) 末梢血単核細胞(PBMC)における炎症応答：TNF-α、MCP-1、CD11c、CD163、HO-1

6) アンケート調査：心身の状態の変化、効果の実感など

| 食事調査 | ○ | ○ | ○ |  |  |
| --- | --- | --- | --- | --- | --- |
| 栄養指導 | ○ | ○ | ○ |  |  |
| 身体計測 | ○ ^1)^ | ○ | ○ | ○ | ○ ^1)^ |
| 血液・尿検査 | ○ ^1)^ | ○ | ○ | ○ | ○ ^1)^ |
| 血管機能検査 |  | ○ | ○ | ○ |  |
| 食事負荷試験 |  | ○ | ○ | ○ |  |
| アンケート |  |  | ○ | ○ | ○ |

^1)^ 診察時の結果を収集する。

図2 評価スケジュール

**５　試験中止の理由**

１）体調が悪化し、試験を中止して治療を実施する必要があると判断した場合。

２）被験者の都合により、試験継続が困難になった場合。

３）試験食品の摂取が困難になった場合。

４）その他、担当医師が判断した場合。

試験中止は担当医師の総合的な判断によるが、その場合には中止理由、中止時期を明記し、中止の時点で定められている評価を行う。

**６　試験計画の変更及び中止に関する基準**

試験食に関する新たな情報等により、試験の信頼性確保あるいは被験者の安全性確保のため、試験計画の変更あるいは試験の中止が必要になった場合。

**７　被験者の保護**

**7-1．インフォームドコンセント**

被験者に対し、担当医師または担当歯科医師から下記の事項を説明し、書面による同意を得る。

- 本試験の目的・内容と予想される効果・副作用
- 被験者が本試験に同意しない場合でも不利益を受けないこと
- 被験者が本試験に同意した場合でも随時これを撤回できること
- 本試験の目的以外の被験者データの流用がないことと秘密の厳守
- その他、被験者の人権の保護に関して必要な事項

**7-2．被験者の補償**

試験の実施に際し、不測の事態により被験者に問題が生じた場合は、試験実施機関の設備と技術をもってその治療、治癒に万全を尽くすこことする。

**7-3．データの取り扱い**

本試験で得られた個人のデータは、本試験の目的以外には使用せず、また本試験関係者以外に対して秘密厳守とする。

**８　試験終了時の除外規定**

以下を除外規定とする。

- 担当医師が不適切と判断した場合
- 併用薬物を変更した場合

その他、脱落・除外症例の取扱いについては担当医師の指示に従う。

**９　試験期間**

平成23年 9月（倫理委員会承認後）～ 平成26年3月

**１０　試験施設**

滋賀医科大学医学部附属病院糖尿病内分泌内科

サンスター心身健康道場（食事提供）

SRL（血液検査、尿検査）

**１１　連 絡 先**

**１）試験責任医師**

前川 聡　教授

施設名　滋賀医科大学内科学講座 糖尿病腎臓神経内科

所在地　〒520-2192 滋賀県大津市瀬田月輪町

TEL 077-548-2221

**２）試験担当責任医師**

森野 勝太郎　助教

施設名　滋賀医科大学内科学講座 糖尿病腎臓神経内科

所在地　〒520-2192 滋賀県大津市瀬田月輪町

TEL 077-548-2223

西尾 善彦　教授

施設名　鹿児島大学医歯学総合研究科 糖尿病内分泌内科学分野

所在地　〒890-8520 鹿児島市桜ヶ丘８丁目35-1

TEL 099-275-6478

**３）試験担当者**

近藤　慶子

施設名　滋賀医科大学内科学講座 糖尿病腎臓神経内科

所在地　〒520-2192 滋賀県大津市瀬田月輪町

TEL 077-548-2223

**４）試験依頼者**

石角 篤

施設名　サンスター株式会社 研究開発部

所在地　〒569-1195 大阪府高槻市朝日町3番1号

TEL 072-682-5570

**５）試験記録提出先**

前川 聡　教授

施設名　滋賀医科大学内科学講座 糖尿病腎臓神経内科

所在地　〒520-2192 滋賀県大津市瀬田月輪町

TEL 077-548-2221

**１２　作成年月日**

平成23年9月15日

**改訂年月日**

　　　平成24年2月13日
