# Supplementary material for: Fiber-rich diet with brown rice improves endothelial function in type 2 diabetes mellitus: A randomized controlled trial
Source: PLoS One. 2017 Jun 29;12(6):e0179869. doi: 10.1371/journal.pone.0179869 (PMC5491061; doi:10.1371/journal.pone.0179869)
Supplement: S3 File — (DOC) [file pone.0179869.s006.doc]

Effects of brown rice or white rice on glycemic and lipid metabolism, and vascular function

Clinical Research Protocol

Principal Investigator: Hiroshi Maegawa, M.D., PhD

Department of Medicine,

Shiga University of Medical Science

Version: 23-96-2

Updated Date: February 13, 2012

Approved Date: February 21, 2012

Translated Date: October 16, 2016

Translated by Katsutaro Morino

Clinical Research Protocol

**Effects of brown rice or white rice on glycemic and lipid metabolism, and vascular function**

**1 Background**

We have been reported that high-fiber diet intervention with combination of brown rice and fiber-rich dishes improve endothelial function in patients with type 2 diabetes mellitus (T2DM)^1,2^. However, it remains unknown whether brown rice is an essential factor to improve endothelial function in these patients. In this study we hypothesized that a simple dietary intervention with brown rice will be acceptable dietary intervention in out-patients and improve vascular function through change in glycemic and lipid metabolism.

**2 Study objectives**

To evaluate the effect of brown rice or white rice-based diet intervention on glycemic　and lipid metabolism, and vascular function.

**3 Study design and schedule**

**3.1 Patients**

Patients with T2DM will be invited to participate at the Shiga University of Medical Science Hospital. Shiga, Japan.

**Inclusion criteria:**

1) T2DM aged between 40 and 80 years

2) HbA1c < 8.0% (JDS)

3) Patients who don't use insulin therapy

4) Patients who don't take alpha-glycosidase inhibitor (alpha-GI)

5) no smoking

**Exclusion criteria**

1) Patients with severe vascular, hepatic, renal, cancer, and/or infectious disease

2) Patients who changes drug treatment 2 months before start the study

3) Patients with rapid weight loss

4) Patients who enrolled in another study within 1 month

5) Patients who have eaten brown rice

6) Patients who have taken fiber supplement

7) Pregnancy and breast-feeding

8) Patients who have taken warfarin and NSAIDS

**3.2 Number of patients**

30 patients (15 patients per group, 2 groups) Brown rice group, White rice group

**3.3 Intervention**

Intervention: Brown rice

Control: White rice

**3.4 Intervention procedure**

Both brown rice (for the fiber-rich diet) and white rice will be provided in small single-serve pouches and shipped to subjects for the 8 weeks to limit energy intake. In the brown rice diet group, participants will be instructed to consume brown rice as a staple food for 10 out of 21 meals per week and to maintain energy intake at 28–30 kcal/kg for 8 weeks. Similarly, in the white rice diet group, participants will be instructed to consume white rice as a staple food for 10 out of 21 meals per week and to maintain energy intake at 28–30 kcal/kg for 2 months.

**3.5 Study design and schedule (Figure 1)**

a randomized, open-labeled, parallel-controlled trial

**3.5.1 Run-in period**

Eligible patients undergo a 4- to 8-week run-in period to maintain a stable diet and assess tolerability. Participants will be advised by a dietician to have a standard diet for diabetes at the beginning of the run-in period.

**3.5.2 Intervention period (2 months)**

After the run-in period, participants will be randomly assigned to a brown rice or white rice diet. Both brown rice and white rice will be provided in small single-serve pouches for the 8-week study to limit energy intake. Participants will be instructed to consume provided pouches as a staple food for 10 out of 21 meals per week and to maintain energy intake at 28–30 kcal/kg for 8 weeks.

**3.5.3 Energy control period (2 months)**

After the intervention period, participants will be instructed to maintain energy intake at 28–30 kcal/kg for 8 weeks. During this period, participants will not be allowed to consume brown rice.

**3.5.4 Follow up period (2 months)**

After the energy control period, all participants can have un-restricted diet without dietitian’s advice.


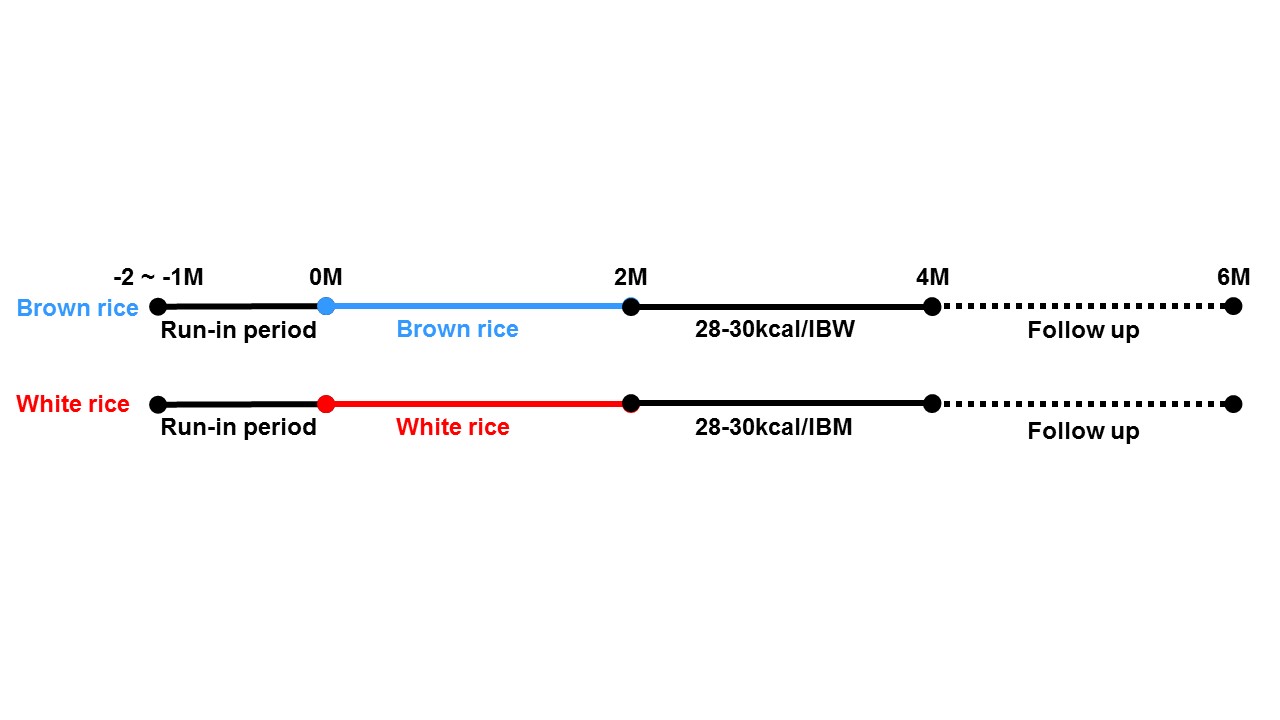
Figure 1 Study schedule

**3.6 Dietary assessment**

During the run-in, intervention, and energy control periods, a dietician will provide participants with written and verbal instructions regarding the completion of a 3-day dietary record with a digital photograph of each meal. A camera will be provided to participants right before dietary assessment.

**3.7 Meal tolerance test**

A rice tolerance test will be performed before and after the intervention. The participants in each group ingests 150 g of brown or white rice. The nutritional composition of both brown rice and white rice will be analyzed soon.

**3.8 Medications (Include supplements)**

During run-in and intervention period (4 months), addition, dosage changes, or discontinuation of any medications should not be performed as a rule. In case of hypoglycemia, dosage changes or withdrawal is permitted. However, a description of the procedure needs to be recorded on the case report form. In addition, any supplements should not be permitted.

**4 Outcomes**

Change in outcomes between 2 month and 0 month. In addition carryover effect will be analyzed at 4 month.

**4.1 Primary outcomes**

Endothelial function

**4.2 Secondary outcomes**

Change in the following data during the intervention period

1) body weight, waist, blood pressure, body fat, abdominal visceral fat

2) AST, ALT, gamma-GTP, HbA1c, glycoalbumin, Total cholesterol, HDL cholesterol,

LDL cholesterol, tPAI-1, hs-CRP, adiponectin, leptin

3) mRNA expression of M1/M2 phenotype of PBMC (TNF-alpha, MCP-1, CD11c,

CD163, HO-1)

4) meal tolerance test (0, 30, 60, 90, 120min): glucose, insulin, triglyceride, free fatty

acid, glucagon, GLP-1, GIP

5) Urine 8-isoprostan, creatinine


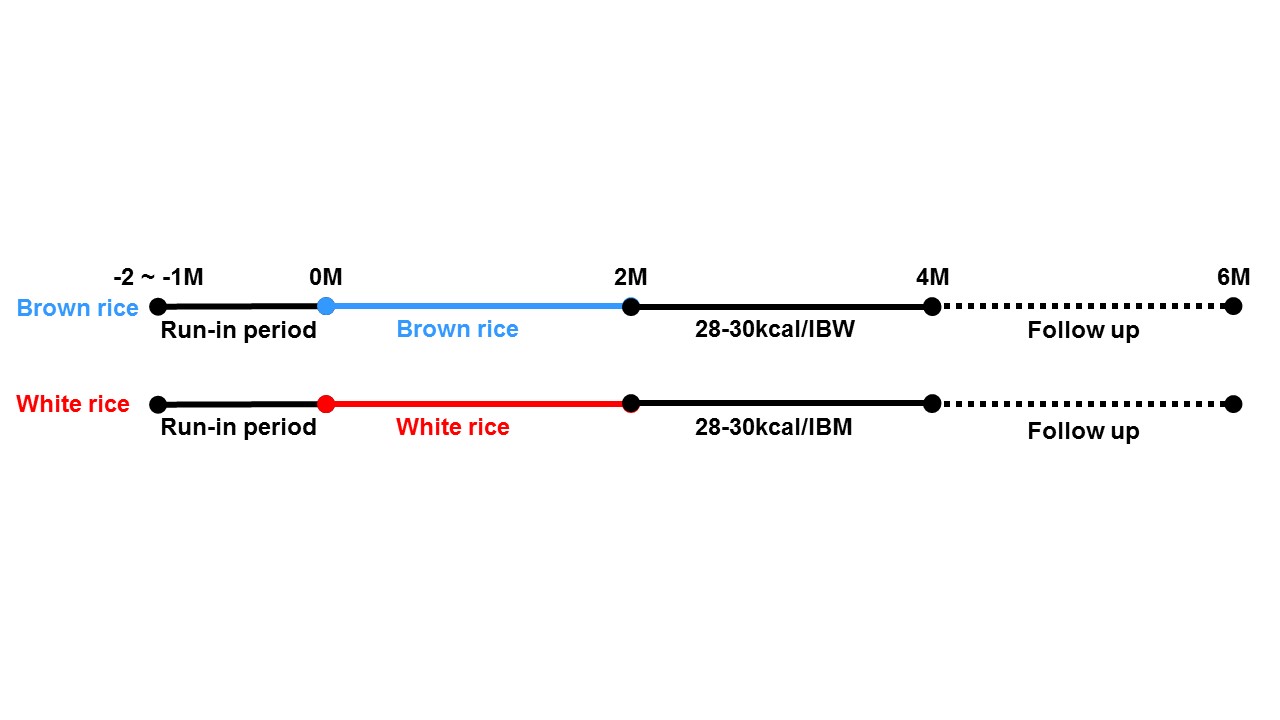
6) change in the eating behavior and psychosomatic state by questionnaire

| Dietary assessment | ○ | ○ | ○ |  |  |
| --- | --- | --- | --- | --- | --- |
| Dietitian guidance | ○ | ○ | ○ |  |  |
| Body weight | ○ ^1)^ | ○ | ○ | ○ | ○ ^1)^ |
| Blood test | ○ ^1)^ | ○ | ○ | ○ | ○ ^1)^ |
| Urine test |  | ○ | ○ | ○ |  |
| Endothelial function |  | ○ | ○ | ○ |  |
| Meal tolerance test |  | ○ | ○ | ○ |  |
| Questionnaire | ○ | ○ | ○ |  | ○ |

^1)^ These information will be collected from charts.

Figure 2. Data collections

**5 Discontinuation criteria for individual patients**

1. The principal investigator or sub-investigator determine that the study should be discontinued due to sick days etc.
2. Withdrawal of consent
3. Difficulty in continuation of dietary intervention
4. The other reasons which principal investigator or sub-investigator decide

The principal investigator or sub-investigator needs to report a reason and exact date of discontinuation, and collect basic information at this time point.

**6 Discontinuation and interruption of study**

The principal investigator should determine whether continuation of the study can be justified when significant information regarding quality, safety are newly recognized.

**7 Human rights of patients and consideration for safety and disadvantages**

**7.1 Informed consent**

Prior to the start of clinical research, the principal investigator should fully inform patient candidates all pertinent aspects of the clinical research by using the informed consent form which has been approved by the ethical review committee. Written informed consent from all participants are required.

1) Objectives, significance, potential risks and benefits of the research

2) The fact that the refusal or withdrawal of consent by a research subject does not cause any disadvantage to such research subject.

3) Research subjects may withdraw their consent at any time even after they have given consent with regard that the research is commenced or continued.

4) Personal information will be protected with process of anonymization.

5) The other important procedure to protect human rights will be conducted under the guideline of Shiga University of Medical Science.

**7.2 Compensation for health hazards**

The principal investigator and sub-investigator upon recognition of adverse events should perform appropriate treatment immediately and record the incidence in medical records.

**7.3 Handling of personal information**

All personal data for this research will not be used for other than this study and protected.

**8 Per-protocol analysis**

Subjects who have received or discontinued medication may exclude from final analysis.

Decision will be made by sub-principle investigators.

**9 Study Duration**

The results of this study will be published at medical journal and/or annual meeting of scientific association. Personal information will be protected also at this moment.

**11 Research Institutions**

**11.1 Medical Institutions**

　　Shiga University Hospital

**11.2 Provider of test meal**

　　Sunstar Inc.

**11.3 Clinical laboratory services**

　　SRL, Inc (blood, urine)

Techno Suruga Lab (micro flora)

JCL Bioassay Corporation (lipid metabolites)

**12 Funding**

This study will be funded by the promotion grants to the Department of Medicine, Shiga University of Medical Science (Shogaku-kifu), and Sunstar Inc.

**13 Conflict of Interest**

Potential conflict of interest will be reported based on the instruction of journal and association.

**14 Research organization**

**14.1 Principle investigator**

Hiroshi Maegawa, MD, PhD

Professor

Department of Medicine, Division of Endocrinology and Metabolism, Nephorology, Neurology

Shiga University of Medical Science

Seta-Tsukinowa-Cho, Otsu, Shiga 520-2192, Japan

Tel.: +81-77-548-2221

**14.2 Principle investigator for implementation**

Katsutaro Morino, MD, PhD

Assistant Professor

Department of Medicine, Division of Endocrinology and Metabolism

Shiga University of Medical Science

Seta-Tsukinowa-Cho, Otsu, Shiga 520-2192, Japan

Tel.: +81-77-548-2223

Yoshihiko Nishio, MD, PhD

Professor

Department of Diabetes and Endocrine Medicine,

Kagoshima University Graduate School of Medical and Dental Sciences

8-35-1 Sakuragaoka, Kagoshima, 890-8520, Japan

Tel.: +81-99-275-6478

**14.3 Sub-investigator**

Keiko Kondo, PhD

Department of Medicine, Division of Endocrinology and Metabolism

Shiga University of Medical Science

Seta-Tsukinowa-Cho, Otsu, Shiga 520-2192, Japan

Tel.: +81-77-548-2223

**14.4 Contact information for cooperative research**

Atsushi Ishikado, PhD

R&D Department

Sunstar Inc.

3-1, Asahicho, Takatsuki, Osaka 569-1195, Japan

Tel.: +81-72-682-5570

**14.5 DATA office**

Hiroshi Maegawa, MD, PhD

Department of Medicine, Division of Endocrinology and Metabolism, Nephorology, Neurology

Shiga University of Medical Science

Seta-Tsukinowa-Cho, Otsu, Shiga 520-2192, Japan

Tel.: +81-77-548-2221

**15 References**

1. Yoshihiko Nishio, Keiko Kondo, Keiko Nakao, Tetsuya Hashimoto, Atsushi Ishikado, Satoshi Ugi, Atsunori Kashiwagi, Hiroshi Maegawa. A High-Fiber Diet Improves the Vascular Function as Well as Glycemic Control in the Patients with Type 2 Diabetes Mellitus. Diabetes. 2011; 60 (supl1): A110.

2. Keiko Kondo, Yoshihiko Nishio et al. A high-fiber, low-fat diet improves endothelial function in the patients with type 2 diabetes mellitus: a pirot study. Journal of the Japan Diabetes Society. 2011; 54(3): 232, in Japanese

**16 Date of protocol fixation**

September 15, 2011

Updated date:

　　　February 13, 2012
