# Supplementary material for: Fiber-rich diet with brown rice improves endothelial function in type 2 diabetes mellitus: A randomized controlled trial
Source: PLoS One. 2017 Jun 29;12(6):e0179869. doi: 10.1371/journal.pone.0179869 (PMC5491061; doi:10.1371/journal.pone.0179869)
Supplement: S4 File — (DOC) [file pone.0179869.s007.doc]

**研究実施計画書**

**玄米食および白米食が糖・脂質代謝および血管機能に及ぼす影響**

**1 背景**

我々は、2型糖尿病患者において玄米菜食メニューを基本としたレトルト試験食（玄米ごはん・レトルト惣菜）を用いた食事介入により血糖コントロールおよび血管拡張機能を改善することを報告した ^1,2^。本研究は、より日常生活に即した食事療法を提案するため、主食のみを玄米あるいは白米に置き換えることが、糖・脂質代謝および血管機能などの指標に対する影響について検討する。

**2 目的**

2型糖尿病患者に対して、主食を玄米あるいは白米とする栄養指導を行い糖・脂質代謝および血管機能へ及ぼす影響について検討する。

**3 試験方法**

**3.1　対象**

滋賀医科大学医学部附属病院に通院中の2型糖尿病患者

選択基準：

①40歳以上80歳未満の患者

②HbA1c＜8.0％の患者

③インスリン治療していない患者

④α-グルコシダーゼ阻害薬を服用していない患者

⑤非喫煙者

除外の基準：

①重症の血管病患者、重症の肝・腎障害患者、癌患者、感染症患者

②同意取得直前（2ヶ月以内）に糖尿病薬を変更した者

③急激な体重変化(±10%)を起こした者

④1ヶ月以内に他の臨床試験に参加した者

⑤普段の食事で玄米を摂取している者

⑥食物繊維サプリメントを摂取している者

⑦妊婦、授乳婦、その他担当医師が不適切と判断した者

（現在使用している薬剤は基本的に継続するが、過去1ヶ月以内に抗炎症薬・ワーファリンを使用している場合には除外する。）

**3.2 目標症例数**

目標症例数　30名　（玄米群：15名、白米群：15名）

**3.2.1 目標症例数設定の根拠**

以前我々が報告したデータ ^1,2^より、玄米菜食期間もしくは通常食摂取期間（コントロール期間）前後のFDR *の変化率の平均値は、それぞれ83.1%、19.5%、標準偏差は60.2、40.9であった。これらのデータを用いα＝0.05、1－β＝0.80（危険率5％、検出力80％）の条件下でサンプル計算を行うと22 (11＋11)例となる。本研究は主食のみの変更であり介入条件が異なること、脱落症例等を考慮し30 (15＋15)例を目標症例数と設定する。

* flow debt repayment: 血管拡張機能の評価項目の一つである。

**3.3 試験食**

試験食：玄米ごはん

対照食：白米ごはん

**3.4 摂取方法**

試験食品を宅配し、玄米ごはんあるいは白米ごはんを2回/日、5日/週摂取する。摂取エネルギーは、現行の栄養指導に従い、主食を試験食品へ置き換える。個人毎の摂取エネルギーの管理は、提供する主食の量およびその他の食事で調整する。実施期間は2ヶ月間とする。

**3.5 試験デザインおよび試験期間**

**3.5.1 試験デザイン**

本試験のデザインは、ランダム化比較試験（RCT：Randomized Controlled Trial）とし、被験者を玄米群または白米群に無作為に割り当てる。

**3.5.2 試験期間**

倫理委員会承認後～2014年3月31日

**3.6 試験スケジュール（図１）**

**3.6.1 エネルギー調整期間 (試験開始前1～2ヶ月間)**

試験開始前にエネルギー調整期間を設け、通常の栄養指導を行う。

**3.6.2 玄米または白米食期間 (2ヶ月間)**

エネルギー調整期間終了後、被験者を玄米群あるいは白米群に無作為に割り当てる。玄米群は玄米ごはん、白米群は白米ごはんを宅配にて提供し、主食を置き換える。摂取回数は1日2回、週に5日間とする。主食量は、各個人の設定エネルギー量により調整する。

**3.6.3 エネルギー調整期間 (2ヶ月間)**

玄米または白米食期間後、3.6.1の期間と同様、エネルギー調整期間を設ける。なお、主食は提供しない。この期間、玄米の摂取は不可とする。

**3.6.4 フォローアップ期間 (試験終了後約2ヶ月間)**

3.6.1～4の期間が終了した後、フォローアップ期間を設ける。この期間中の栄養指導は行わず、自由摂取とする。

＊なお、全試験期間中を通して食事内容は糖尿病学会の発行する糖尿病治療ガイドラインに沿ったものとし、摂取エネルギー量は標準体重1kgあたり28～30kcalとする。

図1 試験デザイン

**3.7 食事調査**

　　試験開始前・玄米または白米食期間中(0～2ヶ月)・エネルギー調整期間中(2～4ヶ月)に3

日間の食事調査（記録・カメラ撮影）を行い、栄養摂取量を評価する。なお、食事調査で用いるカメラは事前に患者に渡す。

**3.8 食事負荷試験**

食事負荷試験は、0、2、4ヶ月に実施する。玄米群は玄米、白米群は白米負荷試験を行う。負荷量は各々250kcalとする。表1に栄養分析結果を示す。

表1 玄米・白米の栄養分析結果

(100gあたり)

|  | 玄米 | 白米 |
| --- | --- | --- |
| エネルギー (kcal) | 115 | 155 |
| たんぱく質 (g) | 2.5 | 2.6 |
| 脂質 (g) | 1.0 | 0.4 |
| 炭水化物 (g) | 23.2 | 35.2 |
| 食物繊維 (g) | 1.8 | <0.5 |

（日本食品分析センター）

**3.9 併用薬（ビタミン剤等も含む）**

試験期間（4ヶ月間）中の治療薬および投与量の変更は、原則として認めない。ただし、試験期間中に空腹時血糖値が110以下となった場合など、医師の判断により投与量の変更も可とし、ケースカードに記入する。また、試験期間中、健康食品としてのサプリメントなどの摂取を禁止する。

**3.10 謝礼**

　 フォローアップ期間終了後、すべての患者に玄米ごはんを渡す。

**3.11 割付方法**

　　最小化法にて無作為割付を行う。割付調整因子は、性（男 vs 女）、年齢（66歳以上 vs

　　66歳未満）、BMI（24以上 vs 24未満）とする。

**4 評価項目**

以下の臨床評価項目について各群における0, 2ヶ月の変化および4ヶ月のキャリーオーバー効果について評価する。

**4.1 主要評価項目**

血管拡張機能（食事負荷試験前および120分後）

**4.2 副次評価項目**

1) 身体計測：身長、体重、腹囲、血圧、体脂肪、内臓脂肪面積 (dual scan)

2) 頚動脈エコーによるIMT（Intima Media Thickness）*0ヶ月のみ測定

3) CAVI（Cardio Ankle Vascular Index）

4) 血液検査：赤血球、白血球、Hb、Ht、血小板数、AST、ALT、γ-GTP、HbA1c、GA、T-cho、HDL-cho、LDL-cho、tPAI-1、高感度CRP、アディポネクチン、レプチン、ADMA、リピドローム解析

5) 末梢血単球における炎症応答：TNF-α、MCP-1、CD11c、CD163、HO-1

6) 食事負荷試験：（0, 30, 60, 90, 120分）血糖、血清インスリン、TG、FFA、

　　　　　　　　 （0, 30, 60, 120分）グルカゴン、GLP-1、GIP

7) 尿検査：8-イソプロスタン、クレアチニン

8) 便検査：腸内細菌叢（フローラ）解析

9) アンケート調査：食行動、心身状態の変化、効果の実感など

| 食事調査 | ○ | ○ | ○ |  |  |
| --- | --- | --- | --- | --- | --- |
| 栄養指導 | ○ | ○ | ○ |  |  |
| 身体計測 | ○ ^1)^ | ○ | ○ | ○ | ○ ^1)^ |
| 内臓脂肪面積測定 |  | ○ | ○ | ○ |  |
| IMT |  | ○ |  |  |  |
| CAVI |  | ○ | ○ | ○ |  |
| 血液検査 | ○ ^1)^ | ○ | ○ | ○ | ○ ^1)^ |
| 尿検査 |  | ○ | ○ | ○ |  |
| 便検査 |  | ○ | ○ |  |  |
| 血管機能検査 |  | ○ | ○ | ○ |  |
| 食事負荷試験 |  | ○ | ○ | ○ |  |
| アンケート | ○ | ○ | ○ |  | ○ |

^1)^ 診察時の結果を収集する。

図2 評価スケジュール

**5 検体の保存**

以下に示す検体を試験終了後5年間, －80℃にて凍結保存する。これらの検体は、糖尿病関連の何らかの研究に用いる可能性がある。この場合、当該研究について公表し対象者が拒否できる機会を提供する。

1) 血清 約1ml×5本 *

2) 血漿 約1.5ml×2×5本 *

3) 尿 約2ml×3本

* 食事負荷試験前後の5pointで採取した検査用血液の余剰分より分離し、保存用に採血することはない。

**6 統計学的処理**

血管拡張機能の評価項目である最大血流増加率 (peak FBF; forearm blood flow), 血流増加持続時間, 血管内皮機能の指標であるFDR (flow debt repayment)について各群の0,2,4Mのpaired t-test、2群間の0,2Mの変化率のunpaired t-testを行う。

**7 試験中止の理由**

１）体調が悪化し、試験を中止して治療を実施する必要があると判断した場合。

２）被験者の都合により、試験継続が困難になった場合。

３）試験食品の摂取が困難になった場合。

４）その他、担当医師が判断した場合。

試験中止は担当医師の総合的な判断によるが、その場合には中止理由、中止時期を明記し、中止の時点で定められている評価を行う。

**8 試験計画の変更及び中止に関する基準**

試験食に関する新たな情報等により、試験の信頼性確保あるいは被験者の安全性確保のため、試験計画の変更あるいは試験の中止が必要になった場合。

**9 被験者の保護**

**9.1 遵守すべき諸原則**

　本研究は「ヘルシンキ宣言」および「臨床研究の倫理指針」（厚生労働省　平成20年7月31日改正）に則って行う。

**9.2 倫理委員会**

　本研究は滋賀医科大学倫理委員会で承認を得て行う。

**9.3 インフォームドコンセント**

被験者に対し、担当医師または担当歯科医師から下記の事項を説明し、書面による同意を得る。

1) 本試験の目的・内容と予想される効果・副作用

2) 被験者が本試験に同意しない場合でも不利益を受けないこと

3) 被験者が本試験に同意した場合でも随時これを撤回できること

4) 本試験の目的以外の被験者データの流用がないことと秘密の厳守

5) その他、被験者の人権の保護に関して必要な事項

**9.4 被験者の補償**

試験の実施に際し、不測の事態により被験者に問題が生じた場合は、試験実施機関の設備と技術をもってその治療、治癒に万全を尽くすこことする。

**9.5 試験に係る費用**

本試験で実施する全ての検査費用および提供する主食の費用は研究費で負担する。

**9.6 データの取り扱い**

本試験で得られた個人のデータは、本試験の目的以外には使用せず、また本試験関係者以外に対して秘密厳守とする。

**10 研究成果の公表**

本研究で得られた結果は学会または論文として公表される。その際、被験者の個人情報

が出ることはない。

**11 試験施設**

**11.1 実施施設**

　　滋賀医科大学医学部附属病院

**11.2 食事提供施設**

　　サンスター心身健康道場

**11.3 検査依頼施設**

　　SRL（血液・尿検査）

　　テクノスルガラボ（便検査）

　　JCLバイオアッセイ（リピドローム解析）

**12 研究費**

滋賀医科大学委任経理金およびサンスター研究費

**13 利益相反**

結果を公表する学会および雑誌の規定に準じて報告する。

**14 連絡先**

**14.1 試験責任医師**

前川 聡　教授

施設名　滋賀医科大学内科学講座 糖尿病腎臓神経内科

所在地　〒520-2192 滋賀県大津市瀬田月輪町

TEL 077-548-2221

**14.2 試験担当責任医師**

森野 勝太郎　助教

施設名　滋賀医科大学内科学講座 糖尿病腎臓神経内科

所在地　〒520-2192 滋賀県大津市瀬田月輪町

TEL 077-548-2223

西尾 善彦　教授

施設名　鹿児島大学医歯学総合研究科 糖尿病内分泌内科学分野

所在地　〒890-8520 鹿児島市桜ヶ丘８丁目35-1

TEL 099-275-6478

**14.3 試験担当者**

近藤　慶子

施設名　滋賀医科大学内科学講座 糖尿病腎臓神経内科

所在地　〒520-2192 滋賀県大津市瀬田月輪町

TEL 077-548-2223

**14.4 試験依頼者**

石角 篤

施設名　サンスター株式会社 研究開発部

所在地　〒569-1195 大阪府高槻市朝日町3番1号

TEL 072-682-5570

**14.5 試験記録提出先**

前川 聡　教授

施設名　滋賀医科大学内科学講座 糖尿病腎臓神経内科

所在地　〒520-2192 滋賀県大津市瀬田月輪町

TEL 077-548-2221

**15 参考文献**

1. Yoshihiko Nishio, Keiko Kondo, Keiko Nakao, Tetsuya Hashimoto, Atsushi Ishikado, Satoshi Ugi, Atsunori Kashiwagi, Hiroshi Maegawa. A High-Fiber Diet Improves the Vascular Function as Well as Glycemic Control in the Patients with Type 2 Diabetes Mellitus. Diabetes. 2011; 60 (supl1): A110.

2. 近藤慶子, 西尾善彦, 中尾恵子, 橋本哲也, 石角篤, 卯木智, 柏木厚典, 前川聡. 糖尿病患者における低脂肪・高繊維食が血管拡張機能に及ぼす影響. 糖尿病. 2011; 54(3): 232.

**16 作成年月日**

平成23年9月15日

**改訂年月日**

　　　平成24年4月6日
